# Systematic mapping of threats to U.S. beef production

**DOI:** 10.1093/tas/txag143

**Published:** 2026-09-21

**Authors:** Zhihong Xu, Shuai Ma, Anjorin Ezekiel Adeyemi, Jaehyun Ahn, Ashlynn Kogut, Xiting Zhuang, Guadalupe Ceja, Matt Baker, Kent Toyonaga, Tian Chen, Fahmida Husain Choudhury, Abdul Saboor Rahmany

**Affiliations:** Department of Agricultural Leadership, Education and Communications, Texas A&M University, College Station, TX, United States; Department of Agricultural Education, Communications and Leadership, Oklahoma State University, Stillwater, OK, United States; Department of Agriculture, Fort Hays State University, Hays, KS, United States; Department of Agricultural Education Communication, University of Florida, Gainesville, FL, United States; Department of Teaching, Learning and Culture, Texas A&M University, College Station, TX, United States; Department of Agribusiness and Applied Economics, North Dakota State University, Fargo, ND, United States; Department of Animal Science, Texas A&M University, College Station, TX, United States; Department of Agricultural Leadership, Education and Communications, Texas A&M University, College Station, TX, United States; Department of Agricultural Leadership, Education and Communications, Texas A&M University, College Station, TX, United States; Department of Agricultural Leadership, Education and Communications, Texas A&M University, College Station, TX, United States; Department of Agricultural Leadership, Education and Communications, Texas A&M University, College Station, TX, United States; Department of Agricultural Leadership, Education and Communications, Texas A&M University, College Station, TX, United States

**Keywords:** beef supply chain, cross-border threats, production phase, U.S

## Abstract

For the last 65 years, the United States (U.S.) agriculture has been a global leader in total factor productivity (TFP). Within this highly productive and diverse agri-food system, the beef cattle industry stands out distinctly in its operations and for its substantial contributions. After the 11 September 2001, attacks, potential threats to the U.S. agricultural sector, especially the beef supply chain, became a key concern. Agroterrorism and other cross-border threats pose severe challenges to this vital supply chain, raising food and national security concerns. This study aims to comprehensively map potential U.S. beef supply chain threats, focusing on the production phase. The production phase refers to the production phases before harvest: feed production, veterinary services, genetics/breeding, cow-calf operations, background and stocker operations, grass-finishing, and feedlots. The review focuses on three goals: (a) the distribution of research on threats to the production phase of the U.S. beef supply chain. (b) Systematically identify and categorize risks in U.S. beef production, focusing on variables such as threat categories, threat type, specific diseases, pathogens, transmission sources, and their impacts on stakeholders and cattle health. (c) Categorize the solutions/strategies. We set the time frame from 2010 to 2024 and included the publications in the CAB Abstracts (Ovid) database, grey literature from research organizations and conferences proceedings. Evidence and gap maps (EGMs) were employed to visualize the characteristics and distribution of the included studies and identify major scholarly interests and research gaps. This mapping review provides an accessible, comprehensive, and understandable synthesis of data that will inform stakeholders for quickly identifying trends and solutions from diverse research efforts nationally and globally, enabling more effective responses to emerging threats in the U.S. beef supply chain and providing valuable insights for future research to address gaps in the beef production phase.

## Teaser

This review maps the major threats facing early U.S. beef production and highlights where strong evidence exists and where important research gaps remain to support stronger national food and security preparedness.

## Introduction

The U.S. agri-food system remains a global pace-setter for its exceptional efficiency and resilience, seamlessly serving both domestic and international markets while offering a variety of products, especially to citizens at relatively affordable prices (Weersink et al. 2021; Peterson et al. 2023). For the last 65 years, U.S. agriculture has been a global leader in total factor productivity (TFP), which assesses the rate at which total output increases relative to total input growth (USDA 2020). This high level of productivity has led to $2 trillion in yearly revenue, created jobs for 19 million people, both full-time and part-time, and generated $130 billion in profits for over 2.6 million businesses (GAP Initiative 2018; USDA 2020). The primary factors contributing to the rise in TFP include the extensive adoption and effective utilization of advanced seed technologies, precision agriculture, and optimal practices for maintaining soil health, managing nutrients, and ensuring animal well-being (GAP Initiative 2018).

Complementing these achievements, Wang et al. (2015) emphasized the significant impact of agricultural research policies and the supportive research environment in both public and private sectors. They highlighted how extension services and infrastructure, such as roads and irrigation systems, have collectively enhanced the overall productivity of the agricultural sector. Moreover, some authors have argued that more emphasis should instead be placed on aspects such as technical change, total resource productivity, allocative efficiency change, and scale economies (Abramovitz 1956; Gollop and Swinand 1998; Plastina and Lence 2018).

Despite the significant achievements in the sector, various challenges persist. These include labor market issues, environmental concerns linked to groundwater supply, climate change impacts, soil contamination, and atmospheric pollutants, a decrease in rural population, disruptions in the complex agricultural supply chains, and safety concerns regarding genetically modified organisms (GMOs) (Schaible and Aillery 2012, 2017; Wortman and Lovell 2013; Knox et al. 2014; Hatfield and Prueger 2015; Zahniser et al. 2018; Veeck et al. 2020).

Within this highly productive and diverse agri-food system, the beef cattle industry stands out distinctly in its operations and for its substantial contributions. Its pivotal role in U.S. agriculture is well-documented, establishing the country as the leading producer globally (Lowe and Gereffi 2009). Peel et al. (2020) highlighted the significant contributions of the beef cattle industry, noting that as of January 2020, the U.S. had 94.4 million cattle, with over 80 million involved in beef production. The industry’s economic impact was evident for example in 2017, with reported sales of $77.2 billion. Similarly, the sector also encompasses 882,692 farms, including 729,046 dedicated to raising beef cows (Peel et al. 2020).

Cowley (2022) noted the U.S. beef supply chain’s susceptibility to disruptions due to the specific processes involved in raising and slaughtering cattle, further exacerbated by recent changes in the cattle industry that could amplify the long-term impacts of short-term shocks. Moreover, the literature traditionally agrees that beef production comprises four primary segments, reflecting a complex and diverse structure of cow-calf operations, stocker/backgrounding systems, feedlots and meat packers (Albin 1998; Lowe and Gereffi 2009; Yang et al. 2019; Ferdousi et al. 2020; Peel et al. 2020; USDA ERS 2023). In their study, however, Lowe and Gereffi (2009) adopted a different approach by aligning the four primary beef production segments with the broader beef industry value chain. They delineated the segments as follows: inputs (feed, veterinary services, and genetics), production (cow-calf operations, stockers, and backgrounding, feedlots, and dairy beef), processing and distribution (packaging/processing, further processing, and wholesalers), and marketing (supermarkets, restaurants, and foodservice suppliers). The implication of this intricate value chain is the heightened vulnerability peculiar to each phase along the chain to potential or existing threats (domestic or cross-border) if appropriate safeguards are not implemented.

Following the 11 September 2001 attacks, the potential threats to the U.S. agricultural sector, particularly the beef supply chain, became a prominent topic of discussion among stakeholders (Monke 2006; Ashlock et al. 2009, 2012; Riley et al. 2012). Key concerns included the credibility of information sources and awareness levels (Miranowski and Scanes 2008; Riley et al. 2012), the risk of pathogens and the level of preparedness to counter such attacks (Monke 2006), the vulnerability of supply chains (Cupp et al. 2004), and the overall threat to food and national security (Gill 2015).These discussions underscore the critical need for robust strategies to understand these threats, synthesize existing literature on these threats and using these data, develop effective strategies that can protect the nation’s food supply and ensure national security in the event of an agroterrorism attack.

Outside the threat of agroterrorism, numerous other significant threats pose severe challenges to the beef supply chain. Among these, drought stands out as a major concern, potentially devastating feed supplies, as emphasized by Smith (2013). Flooding, too, poses a significant risk, as noted by Crist et al. (2020). The increasing competition for grains, particularly from the expanding biofuel industry, further exacerbates the issue. Environmental concerns, especially regarding greenhouse gas emissions (GHG), have been raised by various stakeholders, with Richter et al. (2020) and Subak (1999) highlighting the beef industry’s substantial environmental impact. Animal rights and welfare issues are also critical, as discussed by Mandel et al. (2022), along with the risks posed by the introduction of foreign animal species such as ticks and tick-borne pathogens (Theuret and Trout Fryxell 2018; Teel and Hairgrove 2024), which can disrupt local ecosystems and agriculture. Additionally, the use of antibiotics and the resulting antimicrobial resistance are significant threats to the industry, as detailed by Cameron and McAllister (2016) and Done et al. (2015). Foodborne illnesses represent another critical issue, with Li et al. (2019) underscoring their impact. Policy and regulatory challenges further complicate the landscape, as explored by Lhermie et al. (2019, 2020) and O’Neill (2005). The effects of climate change also portends significant threats, with Havstad et al. (2018) and Hristov et al. (2018) discussing their potential implications.

Similarly, Gadberry et al. (2019), through a SWOT analysis of the Arkansas beef industry, identified eight key areas of concern: markets and marketing, land availability and cost, technology adoption, cattle genetic merit and health, government regulation, animal activism, labor, and the next generation of producers. These findings underscore the multifaceted challenges facing the U.S. beef industry, highlighting the need for comprehensive strategies to address these risks. Furthermore, the COVID-19 pandemic served as a stark illustration of how unforeseen events can profoundly disrupt the beef supply chain, prompting extensive discussions and analyses. This global crisis highlighted the vulnerabilities within the system, demonstrating the significant and far-reaching impacts that unexpected circumstances can impose on the beef industry (Peel et al. 2020; Balagtas and Cooper 2021; Ramsey et al. 2021; Bina et al. 2022; Hayes et al. 2023).

Considering the profound contribution of the beef industry to U.S. agriculture, the potential or existential threats to this vital supply chain, which can severely disrupt its efficiency, are particularly alarming. It is concerning that, to our knowledge, no comprehensive studies have systematically synthesized the threats to the entire U.S. beef supply chain. While numerous studies have examined individual threats to specific phases within the beef supply chain, a significant literature gap remains in connecting this complex network. This gap makes it challenging for stakeholders to easily access and comprehend the data in an open-access format. An in-depth analysis that traces each phase, identifies connections and sources of threats, assesses transmission risks, evaluates impacts on various stakeholders, and scrutinizes possible solutions and their effectiveness is therefore urgently required.

This pioneering mapping review aims to chart a new course by bridging the existing gap through synthesizing available evidence from published literature over time concerning the threats to the beef supply chain. Initially focusing on all activities preceding the slaughtering of cattle (referred to as the production phase of the beef supply chain), this study will employ a rigorous, transparent mapping review approach, including comprehensive literature searches, critical quality appraisals, and data extraction. By doing so, the team will identify and evaluate various threats, their sources, and their impacts on different stakeholders. The ultimate goal is to provide an accessible, comprehensive, and comprehensible synthesis of data that will inform stakeholders and guide future research and policy development.

### Previous reviews

As previously highlighted, numerous studies have investigated various threats to the U.S. beef supply chain on an individual basis, identifying a wide range of issues but leaving significant gaps in understanding the interrelationships across the entire supply chain. Notably, very few systematic or scoping reviews have specifically synthesized evidence on threats to the U.S. beef supply chain. Most existing reviews either address the broader and more complex agri-food supply chain, analyze it from a global or public health perspective (Penakalapati et al. 2017), focus on single threats, or categorize individual threats into broader groups based on aggregate studies. However, these efforts consistently fall short of providing a comprehensive perspective on the full spectrum of threats impacting the entire supply chain.

In their scoping review aimed at understanding consumer perceptions of antimicrobial use in animal husbandry, particularly given the rising concern of antimicrobial resistance, Barrett et al. (2021) found significant consumer apprehension. Of the 124 studies included, 105 measured consumer concern, with 75% indicating that consumers were worried about antimicrobial use in meat production. The primary reasons for these concerns were human health and animal welfare. The review focused on studies from the United States, Canada, and the European Union (EU), highlighting the widespread nature of these concerns across different regions. Conversely, a study by Sucena Afonso et al. (2024), which included 203 studies, revealed no clear consensus on whether the use or non-use of antimicrobials and their alternatives in livestock production constitutes a threat. While a greater number of their included studies held a positive view, opinions diverged considerably. Other reviews that have researched this threat include those of Landers et al. (2012) and Ma et al. (2019). The interplay between consumer preferences and the demand-supply dynamics presents potential threats to production and management systems, including associated costs within the beef supply chain.

Wiethoelter et al. (2015) conducted an extensive scoping review addressing the threat of infectious diseases at the wildlife-livestock interface. This comprehensive study, which analyzed 15,998 publications spanning from 1912 to 2013, identified the ten most significant diseases in this context: avian influenza, rabies, salmonellosis (excluding *Salmonella abortusovis*), bovine tuberculosis, trichinellosis, Newcastle disease, brucellosis, leptospirosis, echinococcosis, and toxoplasmosis. These diseases are particularly significant for the cow-calf, stocker, and backgrounding segments of the beef supply chain due to the heightened risk of wildlife contact during grazing. Complementing reviews of wildlife-associated disease transmission (Greig et al. 2015; Wiethoelter et al. 2015), Jones et al. (2013) conducted a systematic review examining how agricultural intensification and environmental change influence the emergence of zoonotic diseases at the wildlife–livestock interface. The review identified several examples in which agricultural intensification and environmental change were associated with increased risks of zoonotic disease emergence, partly through the effects of an expanding human population and changing human behavior on the environment. Jones et al. (2013) further concluded that existing research inadequately addresses the interrelated environmental, biological, economic, and social dimensions of zoonotic pathogen emergence, limiting the ability to predict, prevent, and respond effectively to future emergence events.

The implications of these reviews underscore the necessity for a comprehensive, systemic, and holistic approach to synthesizing evidence, such as this mapping study, to identify all potential threats to the U.S. beef supply chain from a systems-approach. This broader perspective is crucial given the significant contribution of the beef industry to U.S. agriculture. This mapping study will provide data-driven evidence that can be easily accessed by various actors and stakeholders across the beef supply chain, where independent operations are interconnected and a threat to one segment can disrupt the entire system. Furthermore, government agencies will find this mapping review invaluable for quickly identifying trends and solutions from diverse research efforts nationally and globally, enabling more effective responses to emerging threats in the U.S. beef supply chain.

### Objectives

This study aims to conduct a comprehensive mapping review of potential threats to the U.S. beef supply chain, focusing on the production phase. The production phase refers to the phases before slaughtering/harvesting. The production phase includes inputs (eg feed/feed production, veterinary services, genetics/breeding) and production (eg cow-calf operations, background operations and stocker operations, grass-finishing, feedlots). The feedlot is the final stage of cattle production. This systematic mapping study has three primary objectives:

1. *Analyze Research Distribution and Gaps*: This mapping review aims to examine the distribution of research on threats to the production phase of the U.S. beef supply chain by analyzing variables such as publication year, country, region, study type, study site, cattle breed, production phase, and cattle production system. The goal is to identify key research gaps and underrepresented areas in the literature.
2. *Identify and Categorize Threats*: This study aims to systematically identify and categorize risks in U.S. beef production, focusing on variables such as threat categories, threat type, specific diseases, pathogens, risk transmissible, disease transmission sources, and their impacts on stakeholders and cattle health. This provides a comprehensive, data-driven reference for stakeholders.
3. *Evaluate and Recommend Mitigation Strategies*: Lastly, this mapping study aims to categorize the solutions/strategies and potentially evaluate the quality of evidence, considering variables like proposed solutions, and documented solution effectiveness. The objective is to provide insights for future research to address gaps and enhance threat management in the beef production phase.

## Materials and methods

### Evidence and gap map: definition and purpose

Evidence and gap maps (EGM) are a synthesis of the available evidence, presented visually and narratively to show the characteristics of evidence related to a research topic (Snilstveit et al. 2013; White et al. 2020). Evidence and gap maps have a broad research question or topic, utilize rigorous and transparent methods for identifying and categorizing literature, and aim to highlight areas where evidence is accessible, areas where gaps in evidence exist, and the overall quality of the existing evidence (James et al. 2016). For this mapping review, we plan to follow the explicit stages of the mapping process outlined by James et al. (2016): (1) Determining the topic and scope; (2) Searching for evidence; (3) Screening evidence; (4) Coding and database development; (5) Reporting and visualizing the findings. As is typical in evidence mapping, our primary aim was to describe the breadth, focus, and design characteristics of the available literature rather than to rate the quality of individual studies. We briefly noted the apparent appropriateness of study designs and analytical methods where relevant, but we did not apply a formal quality appraisal tool or risk-of-bias framework.

### Search strategy

A comprehensive search strategy was developed around the input and production phases of the beef supply chain. To determine the keywords for the database search strategy, the input phase was broken up into feed, veterinary services, and genetics and breeding. The production phase was divided into cow-calf operations, backgrounder/stocker operations, grass finishing, and feedlots. Then, scoping searches were conducted to generate terms to use to describe the threats to each specific subphase. Additionally, ChatGPT (OpenAI, San Francisco, CA, USA) was used to identify additional related keywords. A list of synonyms and related terms were developed for each subphase of the supply chain, for the threats to each subphase of the supply chain, and for beef terms related to that phase. A separate list of terms was created for each of the seven divisions of the production phase of the beef supply chain. Once this list of terms was developed, a beef content expert reviewed the terms and suggested additional terminology to use in the search strategy. Then, an information specialist developed the database search strategy for each of the subphases by combining keywords and applicable subject headings to represent the production subphase, beef, and threats. The keywords were searched in the title and abstract field of the databases. All ten database search strategies are available in the supplementary material A, available as supplementary data at *Translational Animal Science* online.

To assist in the identification of keywords for the grey literature searches, the SR Accelerator’s Word Frequency Analyzer Tool was used to analyze the keywords from a set of studies from the database search marked for inclusion. The grey literature searches utilized the terms identified by the Word Frequency tool as well as keywords from the list of relevant terms, but were not as sophisticated due to limitations in search functionality on the grey literature sites.

### Databases and other sources

Only the CAB Abstracts (Ovid) database was searched due to the volume of literature retrieved by the comprehensive searches. This database covers academic journals and grey literature in the fields of agriculture, veterinary sciences, and environmental studies.

Additional searches were conducted to identify grey literature produced by research organizations focused on the beef production aspects related to U.S. food security. These grey literature sources include:

- Food and Agriculture Organization of the United Nations (FAO), https://www.fao.org/home/en/
- GARDIAN, the Global Agricultural Research Data Innovation & Acceleration Network, https://gardian.bigdata.cgiar.org/#/
- International Livestock Research Institute, https://www.ilri.org/
- USDA National Institute of Food and Agriculture funded grants, https://www.nifa.usda.gov/data/nifa-dashboards/grant-funding-dashboard
- International Food Policy Research Institute (IFPRI) - https://www.ifpri.org/
- Organization for Economic Cooperation and Development (OECD) - https://www.oecd.org/about/
- Google Scholar

Proceedings of the following conferences were searched:

- Society for Veterinary Epidemiology and Preventive Medicine
- American Association of Bovine Practitioners
- North American Veterinary Conference
- American Society of Animal Science

### Selection criteria

For inclusion criteria, the research team identified major concepts that need to be covered which were in alignment with our search strategy: threats to the U.S., production phase, beef supply chain. The inclusion criteria were as follows: (1) The included studies must address/identify specific threat(s) to the production phase of the U.S. beef supply chain, ranging from feed production to the feedlot phase. These threats, including potential threats (eg animal pharmaceutical shortages and diseases), can be biological, environmental, chemical, economic, and so on. (2) The included studies must have been published between (2010) and the present (2024). The timeline was chosen to cover the most recent threats. (3) The included studies should be from peer-reviewed academic literature, governmental and intergovernmental reports (eg OECD, FAO, IFPRI), other important industry and non-governmental organizations (eg veterinary associations) and pre-identified grey literature. (4) The studies should provide some insights into the impact of the identified threats on the U.S. beef supply chain of the production phase. (5) Only studies conducted or reported in English were included. (6) The included studies should be either quantitative or qualitative and provide sufficient methodological information to demonstrate that the research design and analytical methods are appropriate and clearly described. The exclusion criteria were as follows: (1) Studies that do not address or identify specific threats to the U.S. within the production phase of the beef supply chain were omitted. (2) Studies outside the timeframe (2010 to 2024) as well as studies whose date of publishing is difficult to establish, were excluded. (3) Studies that are not peer-reviewed academic literature, governmental or intergovernmental reports, or pre-identified relevant grey literature were excluded to ensure the credibility and reliability of the information. (4) Studies that lack rigorous methodology, including unclear or unsound research designs, methods, and analysis were excluded to maintain the study’s integrity. (5) Studies not reported in English were excluded to maintain consistency and ensure the research team can thoroughly understand and analyze the findings. (6) Review studies and book chapters were excluded.

### Study screening and selection process

After compiling the longlist using EPPI-Reviewer software, our screening process rigorously assessed abstracts and summaries against the predefined inclusion, exclusion, quality, scope, and applicability criteria. This screening involved two distinct groups of reviewers, each consisting of two members, working independently. In case of disagreements, a third reviewer stepped in to resolve any conflicts. Any evidence that did not meet the established criteria were deemed out-of-scope and excluded from further consideration. The result was a refined sub-set of the search database, clearly labeled as “for review.”

Subsequently, we retrieved and examined the full text of all potentially eligible evaluations. This examination was conducted independently by our team members, adhering to our pre-established eligibility criteria. Both inclusion and exclusion decisions required consensus among two reviewers, aligning with Cochrane rapid review methods. This meticulous process ensured inter-rater reliability and the selection of high-quality, pertinent evidence for our mapping study.

Figure 1 presents the PRISMA flow diagram of the screening process. After removing duplicates, the research team screened 66,532 titles and abstracts using the selection criteria., resulting in 56,869 articles being excluded. The authors then reviewed the full text of 9,663 articles. Out of these, 1,064 articles were found to be eligible for the final coding and data analysis.

**Figure 1:**
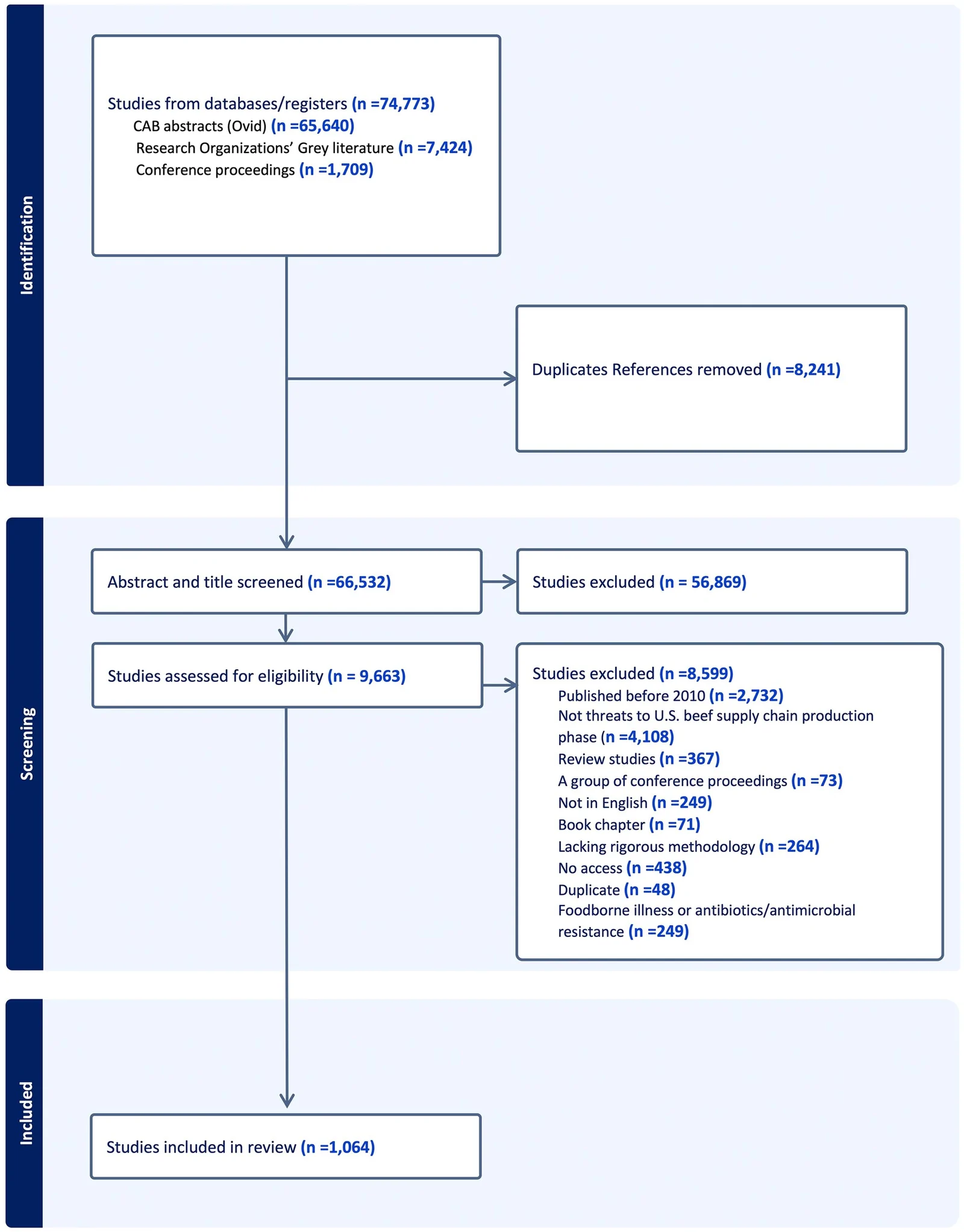
PRISMA flow diagram.

### Coding scheme and data extraction

The coding scheme for the variables of interest are presented below:

Year of Publication**:** The year of publication for the included studies was chosen from 2010 to the present. This period was chosen based on the agreed timeline between the research team and stakeholders to ensure comprehensive coverage of potential risks while also considering the manageable scope of the literature reviewed within the lifetime of the project.

Country and Region: Grouping countries by continent were classified into the following regions: North America, Europe, Asia, Oceania, Middle East, South America, Africa and multi-regional. Although the research primarily focused on the U.S., a regional perspective was incorporated to provide deeper insight into potential or emerging long-term risks that could impact the U.S. beef industry. The country where the study was conducted was included.

Type of study: These were grouped into lab/field testing/pilot study, intervention/diffusion/adoption, observation/case study, model building and theoretical papers, policy studies, and others. These categorizations encompass the most recorded study types in animal sciences while employing a more concise grouping to enhance classification for the sake of our coding scheme. Although they may not represent the highly specific categorizations used by experts in the field of animal science, they have been carefully designed to maintain both scientific rigor and adaptability from an evidence-synthesis standpoint. The framework strives to achieve a balance between systematic organization and the flexibility needed for effective study classification. Additionally, inputs from field experts have been incorporated to refine the approach, though we acknowledge that these categorizations may not fully correspond to statutory classifications in the discipline.

Studies labeled in our coding scheme as lab/field testing/pilot study testing would involve research activities like microbiological cultures, histopathological examinations, molecular diagnostics (eg PCR or sequencing), and immunological assays (eg ELISA) to identify pathogens, understand disease mechanisms as well as evaluating vaccines or drug efficacy (Fulton and Confer 2012). Furthermore, field testing studies, according to our coding scheme, would extend these efforts by monitoring disease outbreaks, assessing vaccine or treatment performance in real-world conditions, and examining environmental factors influencing disease transmission, often through direct observation of livestock or wildlife (Morgavi and Riley 2007). Additionally, pilot studies are those that play a crucial role in testing new diagnostic methods, treatments, or interventions on a small scale before broader implementation, ensuring feasibility and effectiveness, such as evaluating a novel diagnostic tool for pathogen detection (Jorquera-Chavez et al. 2020).

Also, intervention/diffusion/adoption studies, according to our coding scheme, are those that assess the effectiveness of specific strategies, treatments, or practices designed to mitigate or eliminate threats during production, using controlled experiments or observational methods to evaluate their impact (Di Girolamo and Meursinge Reynders 2016), examine the spread of innovations, such as new technologies or management practices, and identify key drivers and patterns that influence their adoption (Montes de Oca Munguia et al. 2021). Furthermore, we coded adoption studies as those that investigate the factors shaping decision-making processes, barriers to implementation, and strategies to enhance the uptake of beneficial innovations (Buchan et al. 2023).

In a similar vein, we interpreted observational studies as those that are essential when experimental studies are impossible or undesirable and are often easier and less expensive to conduct because study subjects and data may already be available or be more easily obtained and are well suited to the analysis of conditions that develop over a long period (Caswell et al. 2018). In observational and case studies, many risk factors or outcomes can be investigated simultaneously, including interactions among variables. Observational studies usually contribute an early foundation of knowledge before it becomes possible to study the disease experimentally (Caswell et al. 2018).

Policy studies, as defined in this study, are those that examine the creation, implementation, and impact of government policies designed to address production threats in the context of beef cattle production. These studies analyze how policies affect producer behavior, production practices, and overall industry outcomes (Lhermie et al. 2020). Model building and theoretical papers, on the other hand, develop new models or frameworks to better understand and address threats to beef cattle production. These studies often use mathematical, statistical, or conceptual models to predict outcomes and provide insights into complex production dynamics (Tedeschi and Menendez 2020; Aherin et al. 2023). Finally, all other study types that do not easily fit into any of the descriptions above were labelled as others.

Study site: state/local, regional, national, global, N/A. This variable was included to provide some form of geographical understanding as to the magnitude of the risks identified from those studies.

Cattle breed. Two separate reviews (Berry et al. 2011; Fernandez-Novo et al. 2020) have highlighted the critical role of cattle breed in determining susceptibility or resistance to various risks within the beef industry. Consequently, this variable was included to document the most commonly raised cattle breeds and to explore potential associations between specific breeds and industry-related risks. We coded as mixed if more than one breed is included in the study (eg Angus and Brahman). Crossbred refers to animals that are the offspring of two or more different breeds.

Production phases: feed/feed production, veterinary services, genetics/breeding, cow-calf operations, background & stocker operations, grass-finishing & feedlots, N/A, mixed, not specified. We strictly followed Lowe and Gereffi (2009) in their analysis of the beef supply chain to establish our working definitions of production phases for this EGM. Studies on feed and feed production examine risks associated with the provision and utilization of essential nutrients—such as forage, grass, grains, supplements, vitamins, and minerals—needed for the proper growth and development of beef cattle at all stages. Veterinary studies focus on key health-related aspects, including vaccinations, parasite management, and the treatment of illnesses and injuries. Additionally, we categorized studies on genetics and breeding as those investigating cattle breed selection, artificial insemination, and genetic testing. Meanwhile, studies on cow-calf operations, backgrounding operations, and grass-finishing and feedlots encompass the three distinct stages of beef cattle farming. These stages involve raising calves until weaning, fattening cattle on pasture, range, and forage, and ultimately confining them to feedlots where they are fed a high-energy grain diet to reach slaughter weight.

Cattle production type/system: feedlot, grazing, not specified, mixed, not applicable (N/A). We categorized the feedlot production system to identify those studies that involve the method of beef cattle production where cattle are confined to a controlled environment and fed a high-energy, grain-based diet to promote rapid weight gain. This system is designed to maximize efficiency and meet market demands for high-quality beef. However, it involves significant management of animal health, environmental impacts, and nutrient use to maintain sustainability (Hubbart et al. 2023). Conversely, studies that involve cattle production systems where cattle feed on natural pasturelands and are allowed to consume a diet more aligned with natural intake are categorized as grazing/pasture-fed systems.

Threat type: These are grouped into unintentional/natural, accidental/non-targeted, and intentional/targeted/bioterrorism. This is aimed at separating risks to beef cattle production systems that are either due to natural occurrences, management errors, or intentionally introduced as acts of bioterrorism.

Threat categories: To develop these categorizations, we first compiled all identified threats from each study. We then applied a systematic and logical classification process to refine and organize them into a concise, structured framework that effectively groups the wide range of identified threats: Biological, Environmental, Management, Nutritional, Chemicals (toxins/pesticides), Economic and market (market fluctuations), Regulatory and compliance, Mixed.

Specific disease: The specific diseases under biological threats are listed.

Pathogens: The specific pathogens causing the diseases under biological threats are listed.

Risk transmissible: These were grouped into yes, no, not mentioned, N/A.

Disease transmission source: animal to animal, animal to human, contaminated environment, mixed, not applicable (N/A), not mentioned.

Threat impact: individual, statewide, national, global

Impact on the cattle’s health: These were grouped into: Respiratory system, Digestion, Reproduction, Musculoskeletal system, Integumentary System, Nervous system, Cardiovascular System, Immune System, Performance, Urinary system, Not specified, Mixed, N/A, Circulatory system, Endocrine system.

Solutions: Vaccination and Disease Control, Policy and Education Initiatives, Environmental and Management Practices, Nutrition and Supplementation, Therapeutic Approaches, Diagnostic and Surveillance Programs, Genetic and Breeding Solutions, Regulatory and compliance, Miscellaneous/mixed, Not specified, N/A.

Effectiveness of the solution: Positive, Negative, not significant, Mixed, N/A, not specified.

### Data coding and management

The coding scheme was collaboratively discussed between the research team and the Advisory Group. To ensure a comprehensive understanding of the coding scheme, the initial ten articles selected for inclusion were coded collectively by the entire research team.

Subsequently, data extraction and coding were carried out independently by two coders. In case of any disagreements, a third‐party arbitrator would step in to facilitate resolution, with the assistance of EPPI‐Reviewer software.

Within the cells of the interactive map, symbols representing the included reviews and primary studies included in the reviews were designed to allow for adjustments in size and/or color. These adjustments enable the exploration of the secondary dimensions described above.

The coded data then was converted to a JSON file, and EPPI‐Mapper was employed to generate the interactive map. Individuals can tap on the matrix in the grid to see a list of related studies, and they can also click on the names of the studies to open the study itself or find more information like references. A link to access the map was generated as part of this process.

## Results

### Year of publication

In 2020 and 2021, the number of studies reached its peak with 95 each (8.9% of the total 1,064). In 2017, 88 studies were published (8.3%). The lowest counts were in 2010 (*n* = 53, 5.0%) and 2024 (*n* = 12, 1.1%). Overall, publications rose steadily from 2010, with more consistent growth after 2015 (Fig. 2).

**Figure 2:**
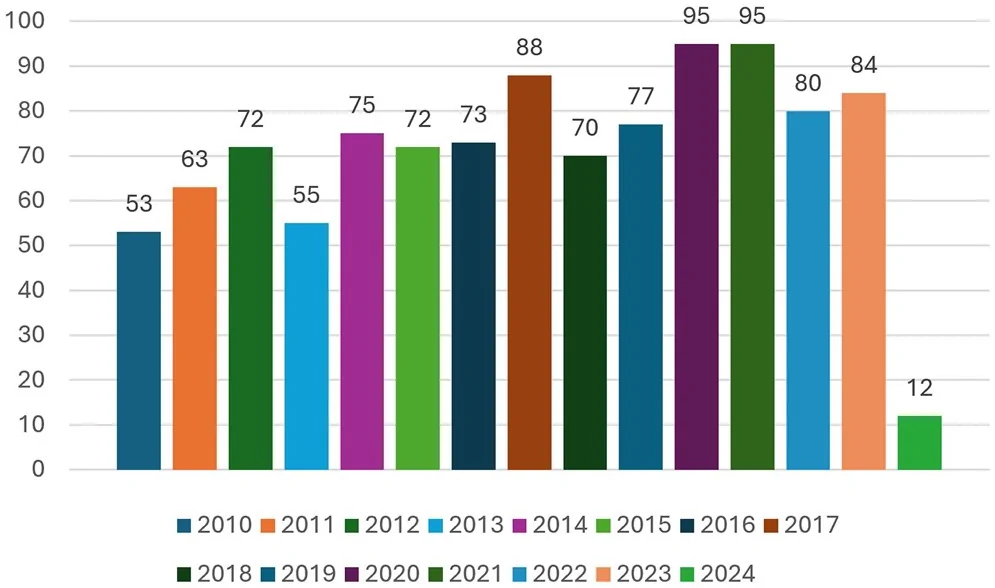
Distribution of studies by year of publication.

### Country and region

Among the 1,064 studies, it is clearly seen that the majority of them originated from the United States, contributing 778 papers (73.1%). However, Canada accounted for the second largest number along with 64 studies (6.0%), followed by Brazil with 26 (2.4%) and Australia with 17 (1.6%). Several other countries, including Germany, Italy, China, France, India, and Japan, each contributed less than 1.6% of the total (Fig. 3). Studies were also reported from a wide range of other countries, but in much smaller numbers. These are where the studies were carried out and not necessarily the distribution of the threats.

**Figure 3:**
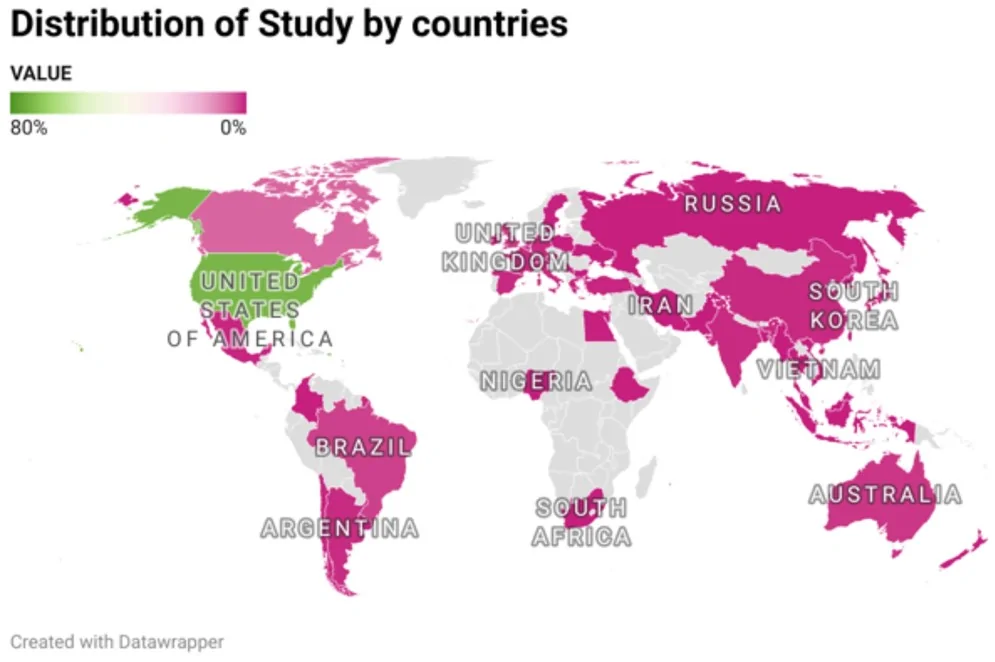
Distribution of studies by countries. An interactive version of this map is available at https://www.datawrapper.de/_/48eZu/.

Of the 1,064 included studies, the vast majority were conducted in North America (*n* = 842, 79.1%). Europe accounted for 91 studies (8.6%), followed by South America with 49 (4.6%) and Asia with 39 (3.7%). Smaller contributions came from Oceania (*n* = 20, 1.9%), Africa (*n* = 9, 0.9%), the Middle East (*n* = 5, 0.5%), and multi-regional studies (*n* = 9, 0.9%) (see Fig. 4).

**Figure 4:**
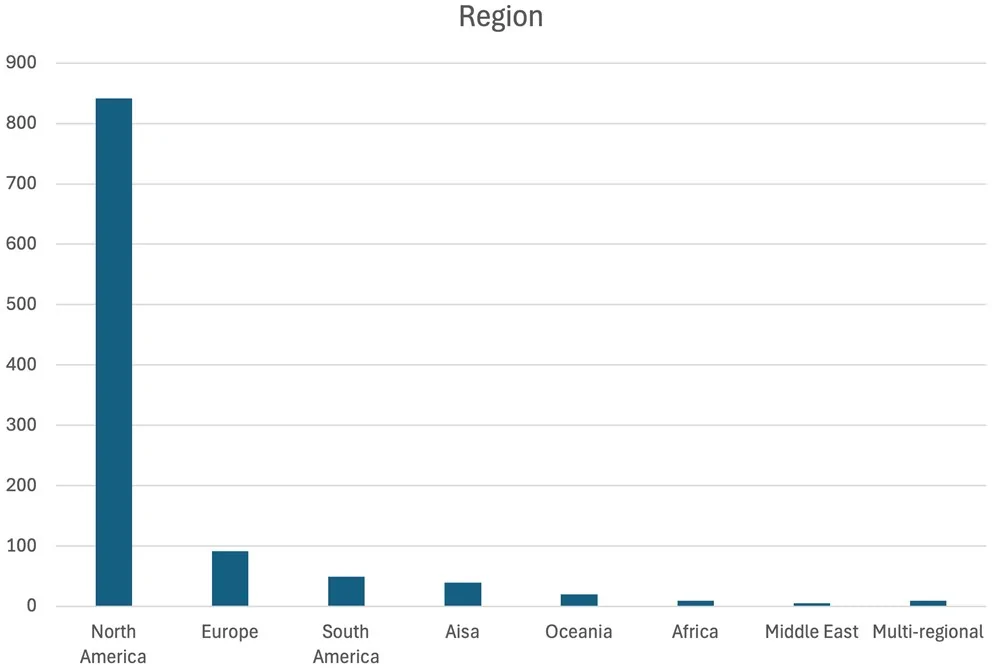
Distribution of studies by region.

### Type of study

As shown in Fig. 5, lab and field testing or pilot studies were the most common type, contributing 59.7% (*n* = 635) of the 1,064 studies. Intervention/diffusion/adoption studies represented 12.7% (*n* = 135), while model building and theoretical papers comprised 12.3% (*n* = 131). Observation or case studies accounted for 11.4% (*n* = 121), followed by others at 2.0% (*n* = 21). Invention studies made up 1.6% (*n* = 17), and policy studies were the least represented at 0.4% (*n* = 4).

**Figure 5:**
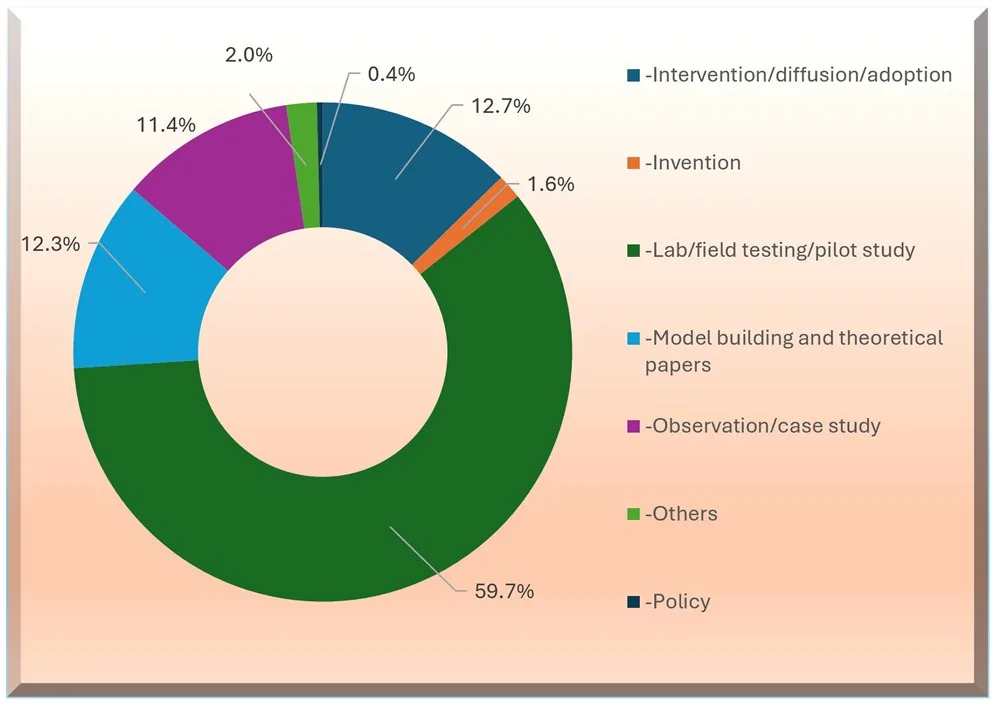
Distribution of studies by type of study.

### Study site

Most studies were conducted at the state or local level (*n* = 645, 60.6%), indicating that risks are frequently assessed within localized or jurisdiction-specific production settings. National-level studies accounted for 16.2% (*n* = 172), followed by regional studies at 13.3% (*n* = 142). A smaller proportion of studies operated at the global scale (*n* = 41, 3.9%), while 6.0% (*n* = 64) did not specify their geographical level (Fig. 6).

**Figure 6:**
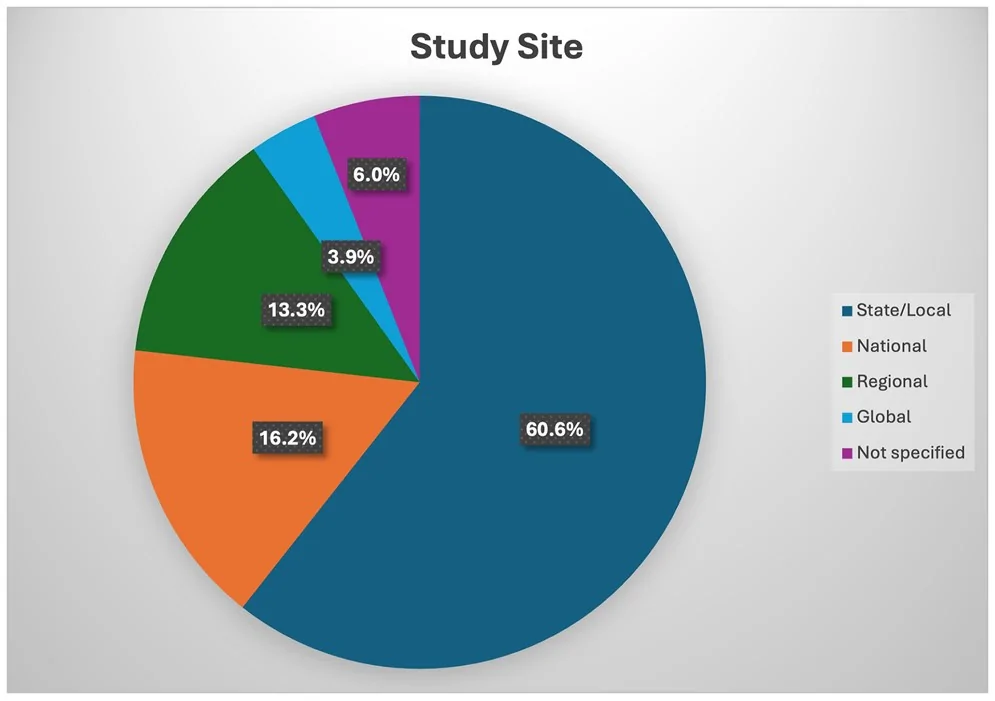
Distribution of study site.

### Cattle breed

Among the 1,064 studies Angus was the most commonly referenced single breed (*n* = 86, 8.1%), followed by Holstein (*n* = 79, 7.4%). A smaller number of studies involved breeds such as Hereford (*n* = 9, 0.8%), Brahman (*n* = 6, 0.6%), Simmental (*n* = 6, 0.6%), Charolais (*n* = 4, 0.4%), and Limousin, Piedmontese, Belgian Blue, and Wagyu, each representing ≤0.2% of the total studies. However, nearly half of the studies (*n* = 486, 45.7%) did not specify any breed and in 112 (10.5%) studies there is no available information about cattle breed category. Additionally, Mixed (more than one breed), and Crossbred cattle mixed breed are reported in 111 (10.4%) studies and 107 (10.1%) studies, respectively (see Fig. 7).

**Figure 7:**
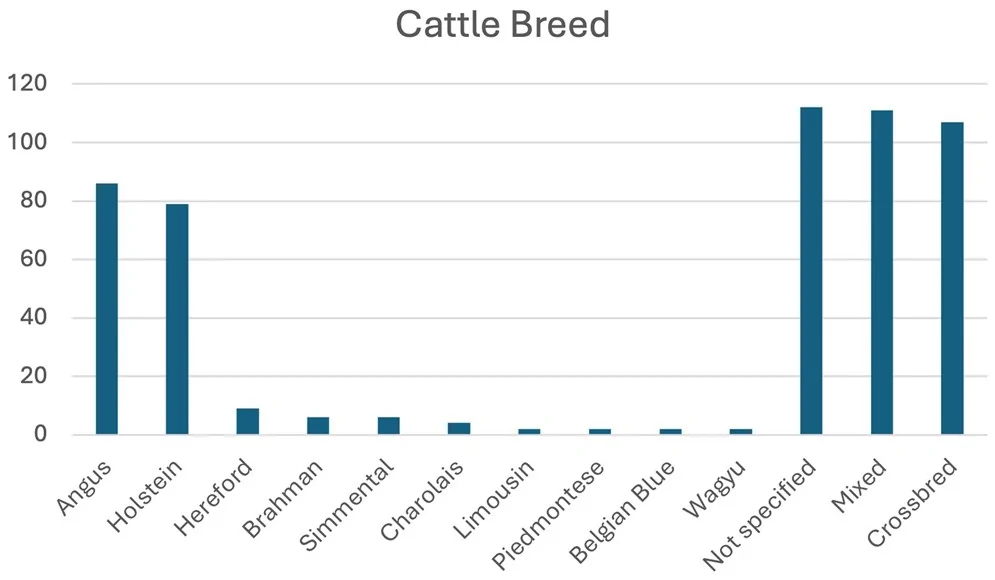
Distribution of cattle breed.

### Cattle production type/system

For cattle production type, feedlot accounted for 33.2% (*n* = 353), grazing for 10.7% (*n* = 114) and mixed systems for 15.4% (*n* = 164). A total of 36.4% (*n* = 387) were categorized as not applicable, and 4.3% (*n* = 46) of the studies did not specify the cattle production system (see Table 1). The data suggested that feedlot is the most common cattle production type, followed by mixed systems and then grazing.

**Table 1:** Cattle production type/system.

Cattle production type/system	*N*	F
**Feedlot**	353	33.2%
**Grazing**	114	10.7%
**Mixed**	164	15.4%
**Not specified**	46	4.3%
**Not applicable (N/A)**	387	36.4%

### Threat type

The majority of the threats were unintentional/natural, accounting for 85.0% (*n* = 904) of the studies. Accidental/non-targeted threats comprised 14.9% (*n* = 159), while intentional/bioterrorism threats were reported in only 0.1% (*n* = 1) of the studies (Table 2). These findings suggest that the unintentional threats are the most common, followed by accidental threats, with bioterrorism being the least common type.

**Table 2:** Threat type.

Threat type	*N*	F
**Unintentional/natural**	904	85.0%
**Accidental/non-targeted**	159	14.9%
**Intentional/targeted/bioterrorism**	1	0.1%

### Threat categories

As demonstrated in Table 3, biological threats were the most common threat category, accounting for 74.5% (*n* = 793), followed by environmental threats (7.8%, *n* = 83) and management threats (*n* = 75, 7.1%). The less common threat included nutritional (3.2%, *n* = 34), chemicals (2.7%, *n* = 29), and economics and market (2.4%, *n* = 26), while mixed threats accounted for 1.9% (*n* = 20). Regulatory and compliance were the least commonly emerging threats, representing only 0.4% (*n* = 4).

**Table 3:** Threat categories.

Threat categories	*N*	F
**Biological**	793	74.5%
**Environmental**	83	7.8%
**Management**	75	7.1%
**Nutritional**	34	3.2%
**Chemicals**	29	2.7%
**Economic and market**	26	2.4%
**Regulatory and compliance**	4	0.4%
**Mixed**	20	1.9%

### Specific disease

The top 20 most frequently mentioned specific diseases are presented in Table 4. Bovine respiratory disease (23.5%, *n* = 250) was the most common disease, followed by foot and mouth disease (FMD) (4.4%, *n* = 47), and bovine viral diarrhea (3.9%, *n* = 41). Other commonly mentioned diseases included cattle toxicosis (2.8%, *n* = 30), Parasite infection (2.7%, *n* = 29), bovine tuberculosis (2.5%, *n* = 27), Johne’s disease (2.2%, *n* = 23), bovine babesiosis (1.7%, *n* = 18), Infertility/pregnancy loss (1.5%, *n* = 16) (see Table 4).

**Table 4:** Top 20 mentioned specific diseases (*n* = 1064).

Specific disease	*N*	F
**Bovine respiratory disease (BRD)**	250	23.5%
**N/A**	218	20.5%
**Not specified**	101	9.5%
**Foot-and-mouth disease (FMD)**	47	4.4%
**Bovine viral diarrhea**	41	3.9%
**Mixed**	30	2.8%
**Cattle toxicosis**	30	2.8%
**Parasite infection**	29	2.7%
**Bovine tuberculosis**	27	2.5%
**Johne’s disease**	23	2.2%
**Bovine babesiosis**	18	1.7%
**Infertility/pregnancy loss**	16	1.5%
**Bovine Spongiform Encephalopathy (BSE)**	15	1.4%
**Liver abscess**	13	1.2%
**Infectious bovine keratoconjunctivitis (IBK)**	12	1.1%
**Bovine anaplasmosis**	12	1.1%
**Cattle fever caused by ticks**	11	1.0%
**Bovine herpesvirus 1 (BHV-1)**	9	0.9%
**Brucellosis**	8	0.8%
**Bacterial bronchopneumonia (BP)**	7	0.7%
**Rift Valley fever (RVF)**	7	0.7%
**Bovine leukosis**	6	0.6%

### Pathogens

The top 20 most frequently mentioned specific pathogens causing the diseases under biological threats are presented in Table 5. Among the included studies, a total of 26.9% (*n* = 286) were categorized as not applicable. 19.0% of the included studies (*n* = 202) didn’t specify the pathogens. Mixed infections were recorded in 139 studies (13.1%). The most frequently identified single agents were bovine viral diarrhea virus (BVDV) and Mannheimia haemolytica (*n* = 46 each, 4.3%), followed by foot-and-mouth disease virus and others (*n* = 27 each, 2.5%), Mycobacterium avium subspecies paratuberculosis (MAP) (*n* = 23, 2.2%), Mycobacterium bovis (*n* = 22, 2.1%), and the cattle tick Rhipicephalus microplus (*n* = 18, 1.7%). Additional agents each accounted for ≤ 1.5% of submissions, including bovine babesiosis due to B. bovis/B. bigemina (*n* = 13, 1.2%), Mycoplasma bovis (*n* = 13, 1.2%), ticks (*n* = 12, 1.1%), Anaplasma marginale (*n* = 11, 1.0%), gastrointestinal nematodes (*n* = 11, 1.0%), bovine herpesvirus-1 (*n* = 10, 0.9%), and a range of bacterial, viral, and parasitic pathogens (each ≤ 0.9%). All remaining identified agents occurred in <10 submissions each (each ≤ 1.0%); examples include Neospora caninum (*n* = 9), BLV (*n* = 8), Brucella abortus (*n* = 8), Histophilus somni (*n* = 7), Moraxella bovis (*n* = 7), E. coli (*n* = 6), Rift Valley fever virus (*n* = 6), MRSA (*n* = 6), BRSV (*n* = 5), F. necrophorum (*n* = 5), and T. foetus (*n* = 5).

**Table 5:** Top 20 mentioned specific pathogens (*n* = 1064).

Specific pathogens	*N*	F
**N/A**	286	26.9%
**Not specified**	202	19.0%
**Mixed**	139	13.1%
**Bovine Viral Diarrhea Virus (BVDV)**	46	4.3%
***Mannheimia haemolytica* (*M. haemolytica*)**	46	4.3%
**Others**	27	2.5%
**Foot and Mouth Disease Virus**	27	2.5%
***Mycobacterium avium* subspecies paratuberculosis (MAP)**	23	2.2%
***Mycobacterium bovis* (Bovine Tuberculosis)**	22	2.1%
***Rhipicephalus microplus* (Cattle tick)**	18	1.7%
**Bovine babesiosis (*B. bovis*, *B. bigemina*)**	13	1.2%
***Mycoplasma bovis* (*M. bovis*)**	13	1.2%
**Ticks**	12	1.1%
***Anaplasma marginale***	11	1.0%
**Nematode (Ostertagia ostertagi/Cooperia oncophora)**	11	1.0%
***Bovine herpesvirus*-1**	10	0.9%
***Neospora caninum***	9	0.9%
**Bovine leukemia virus (BLV)**	8	0.8%
***Brucella abortus* (Brucellosis)**	8	0.8%
***Histophilus somni***	7	0.7%

### Risk transmissible

Of the 1,064 studies analyzed, 56.0% (*n* = 596) were classified as risk transmissible, 20.8% (*n* = 221) did not mention transmissibility. A total of 13.3% (*n* = 141) were categorized as not applicable, and 10.0% (*n* = 106) were considered not transmissible (Fig. 8).

**Figure 8:**
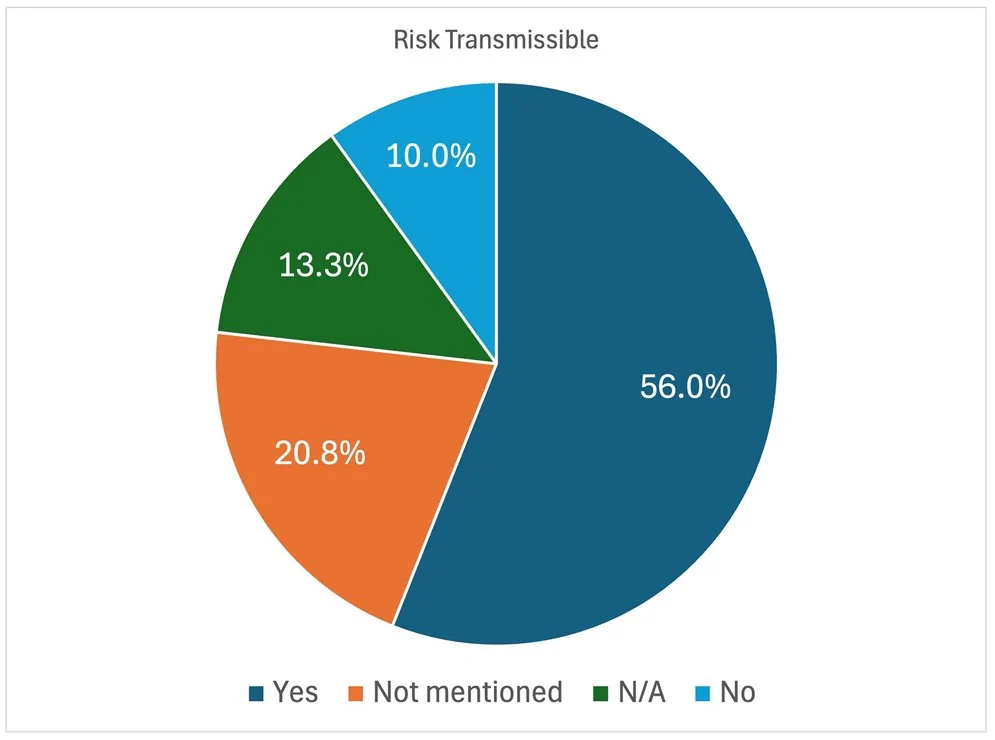
Risk transmissible.

### Disease transmission source

Among the 1064 included studies, the majority reported animal-to-animal transmission (*n* = 393, 36.9%). About 23.5% (*n* = 250) were categorized as not applicable, followed by 21.6% (*n* = 230) that didn’t specify the disease transmission source. There are 92 (8.7%) studies reported mixed sources and 70 (6.6%) in contaminated environments. A smaller proportion involved animal-to-human transmission (*n* = 29, 2.7%), while only a single case (0.1%) reported both animal-to-animal and animal-to-human transmission (Fig. 9).

**Figure 9:**
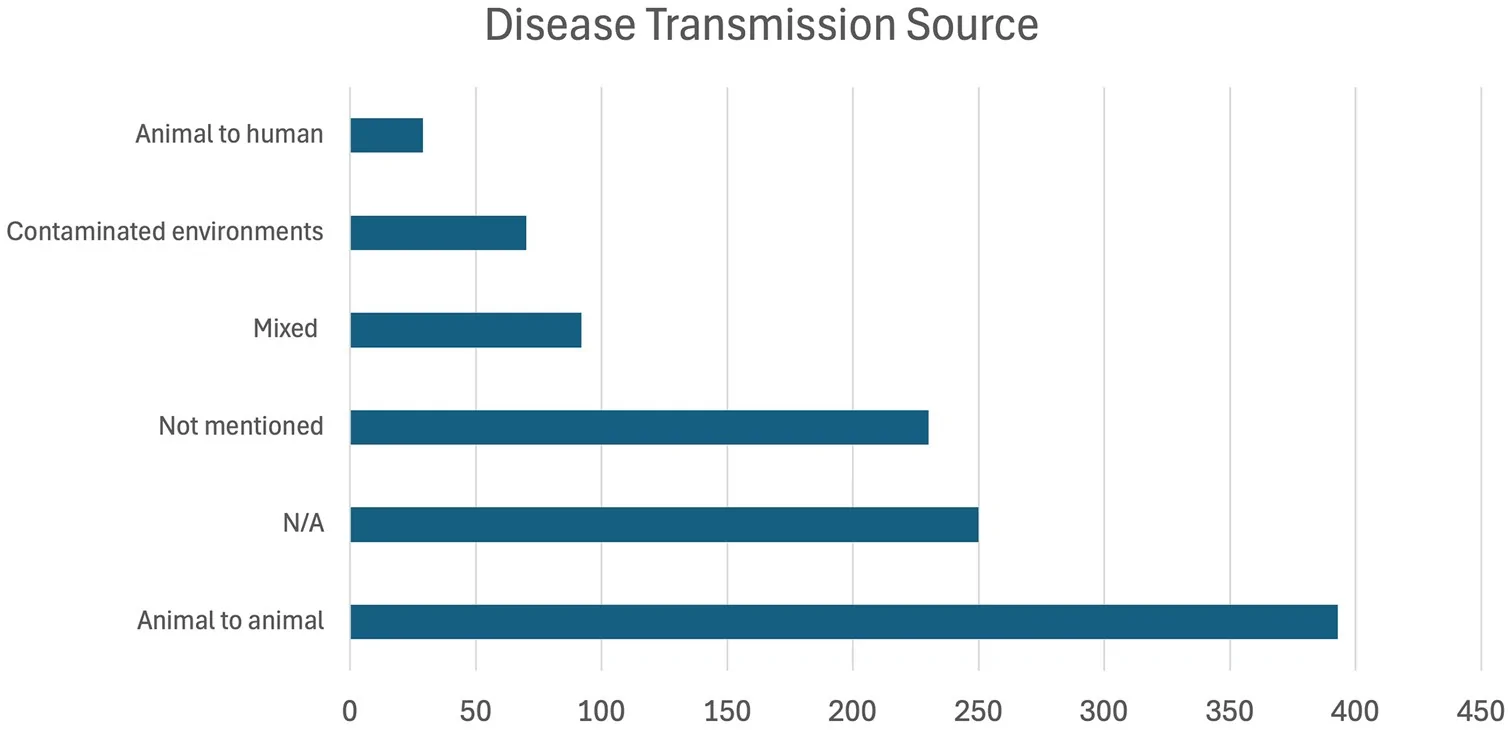
Disease transmission source.

### Threat impact

Among the 1064 studies, most addressed National threats (40.4%, *n* = 430), followed by Global threat (34.8%, *n* = 370). Statewide threat represented (18.0%, *n* = 192), while Individual threat accounted only for (6.8%, *n* = 72). According to the findings, 75.2% of all studies assess threats impact primarily at national and global scales, indicating a predominant focus on broader geographic levels of impact (Fig. 10).

**Figure 10:**
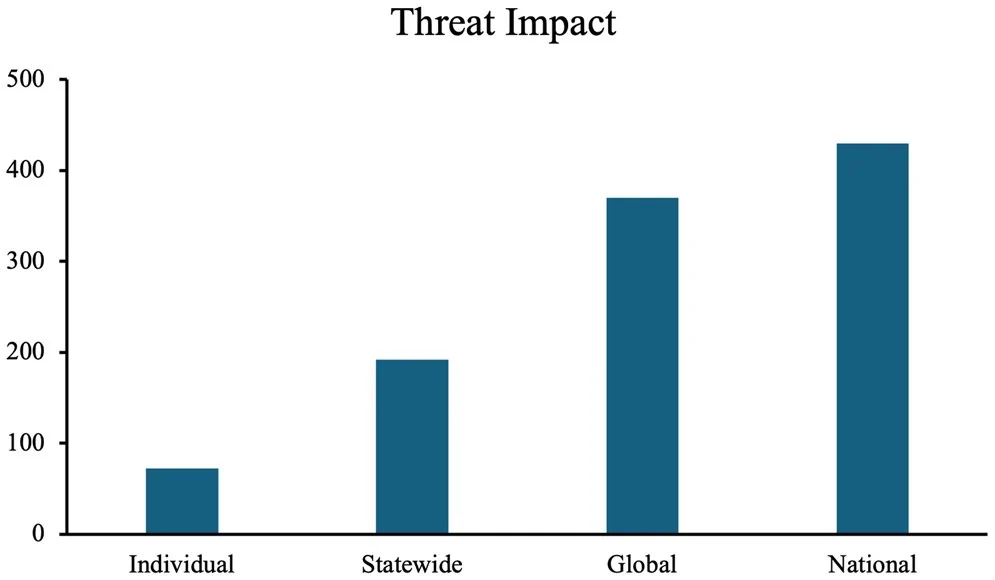
Threat impact.

### Impact on cattle’s health

The impact of threats on cattle’s health is summarized in Fig. 11. The respiratory system was the most frequently reported, accounting for 23.1% of studies (*n* = 246). This was followed by mixed impacts (17.9%, *n* = 190), performance-related impacts (10.1%, *n* = 107), and reproductive impacts (9.0%, *n* = 96). Other health systems such as the digestive, immune, integumentary, nervous, musculoskeletal, and cardiovascular systems together accounted for 20.0% (*n* = 213). Only a very small proportion of studies (0.6%, *n* = 6) addressed threats affecting the circulatory, endocrine, or urinary systems. Finally, 19.4% of studies (*n* = 206) did not specify or did not address health impacts.

**Figure 11:**
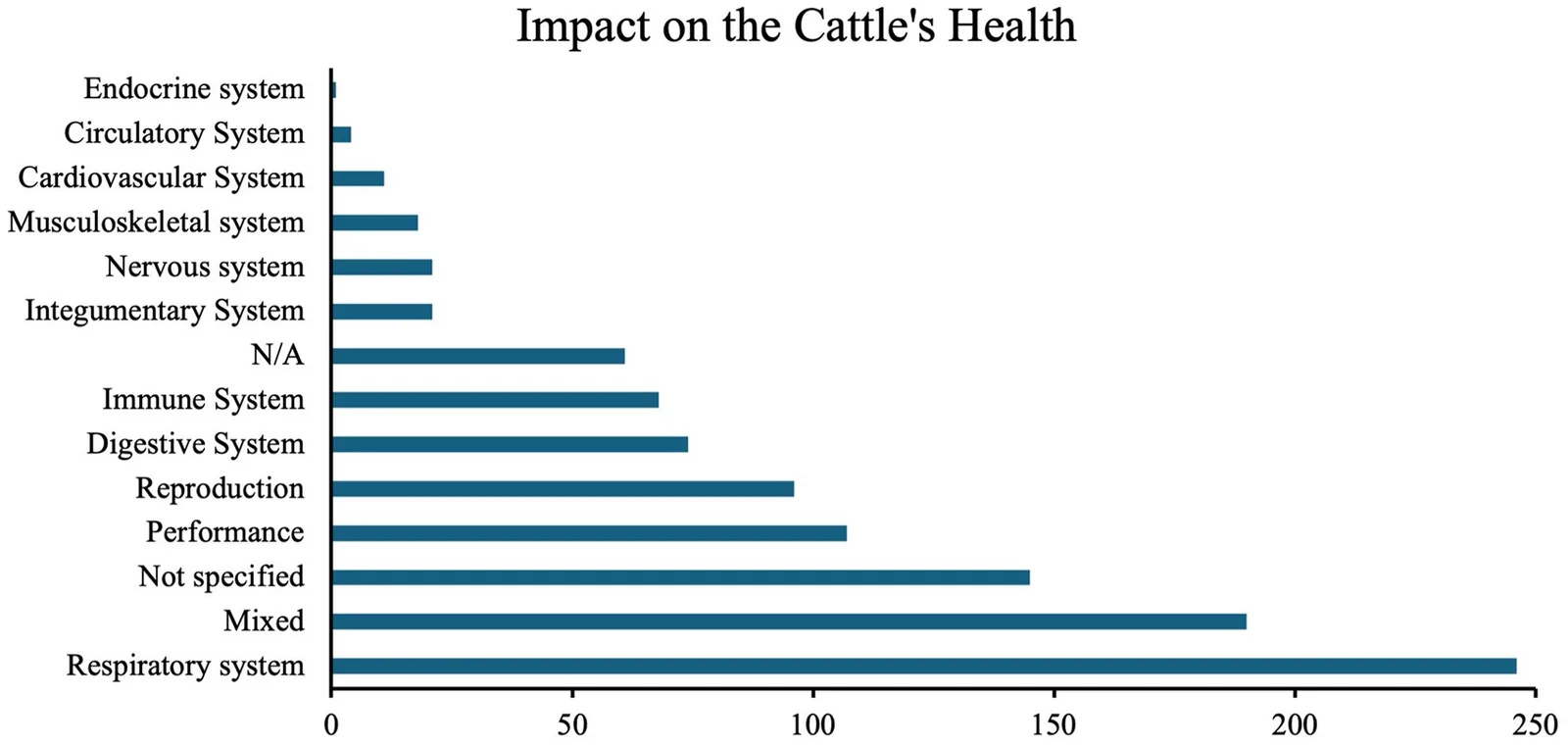
Impact on the cattle’s health.

### Solutions

As shown in Fig. 12, nine types of solutions emerge across studies. The most common was vaccination and disease control (21.2%, 226 studies), followed by diagnostic and surveillance programs (19.5%, 208 studies) and environmental and management practices (14.1%, 150 studies). Other solutions appeared less frequently, including nutrition and supplementation (5.5%), genetic and breeding approaches (5.1%), and therapeutic or surgical approaches (4.6%). Policy and education initiatives (2.5%), mixed/miscellaneous solutions (1.7%), and regulatory/compliance strategies (0.5%) were rarely addressed. Notably, 25.2% of the studies (*n* = 268) did not specify any solution at all.

**Figure 12:**
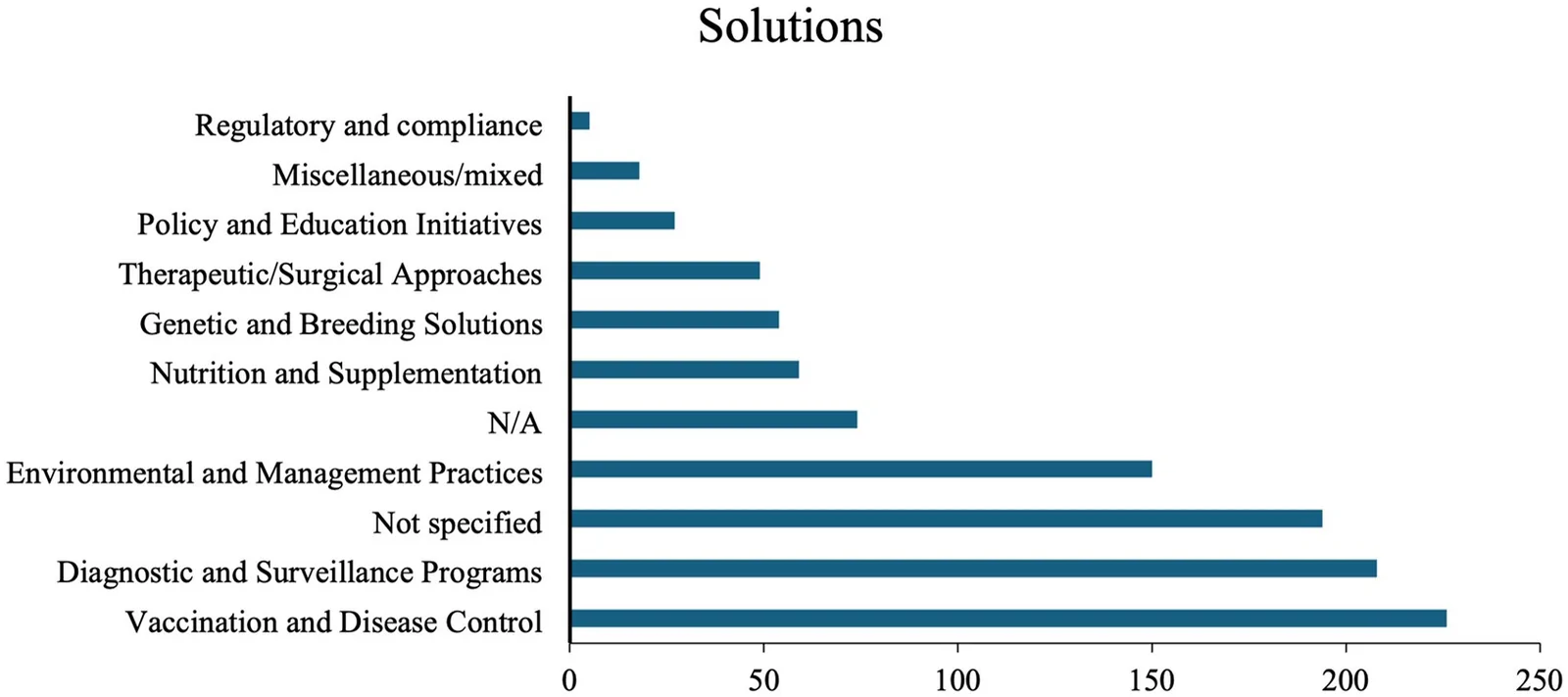
Solutions.

### Effectiveness of solutions

The effectiveness of solutions identified in the studies is summarized in Fig. 13. Almost half of the studies 48.3% (*n* = 514) reported the proposed solution had positive results. In contrast, mixed effectiveness was observed in 7.6% (*n* = 81) of the studies, indicating varying results. A smaller proportion, 4.6%, reported outcomes that were not significant, while just 1.5% reflected negative effectiveness. Notably, 38.0% (*n* = 404) of the studies did not specify or report the effectiveness of the solutions addressed.

**Figure 13:**
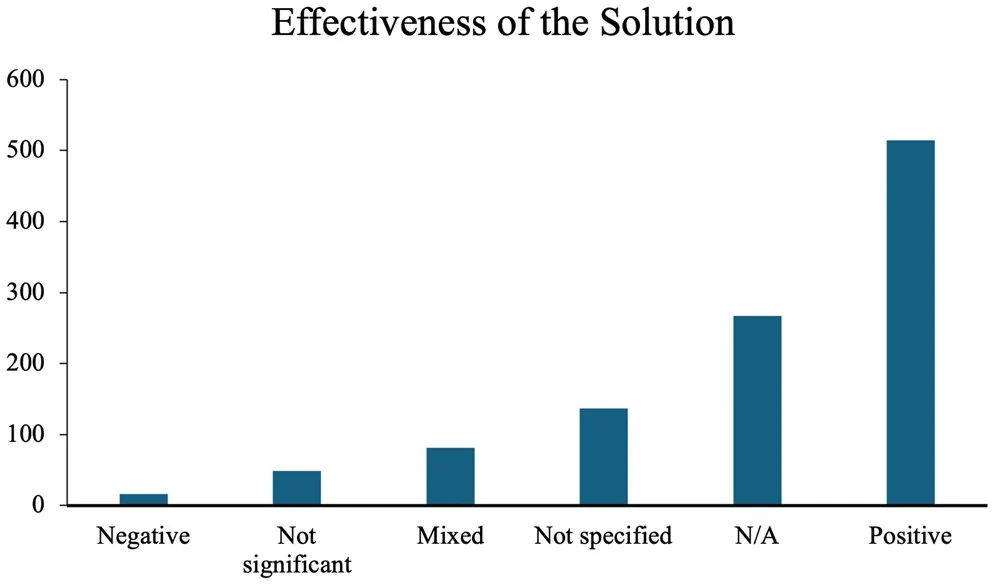
Effectiveness of the solution.

The solutions were grouped into four broad categories illustrated in Fig. 14 plots for their effectiveness. The majority of positive outcomes were reported under medicine and animal health (*n* = 335), followed by farm management (*n* = 153). Almost half of the studies which proposed policy, education and capacity building solutions reported positive outcomes (*n* = 13) and cross-cutting/non-specific approaches (*n* = 13). Negative impacts were rarely reported across all categories, with medicine and animal health (*n* = 12) and farm management (*n* = 4) showing small proportions of negative outcomes, while the other categories reported none. Mixed results were recorded mostly with medicine and animal health (*n* = 47) and farm management (*n* = 32) being most frequent. Similarly, studies that reported no significant effect were largely concentrated in medicine and animal health (*n* = 29) and farm management (*n* = 19). Finally, for the cross-cutting/non-specific, a notable proportion of studies did not report specifically any effectiveness (*n* = 11), and N/A (*n* = 260).

**Figure 14:**
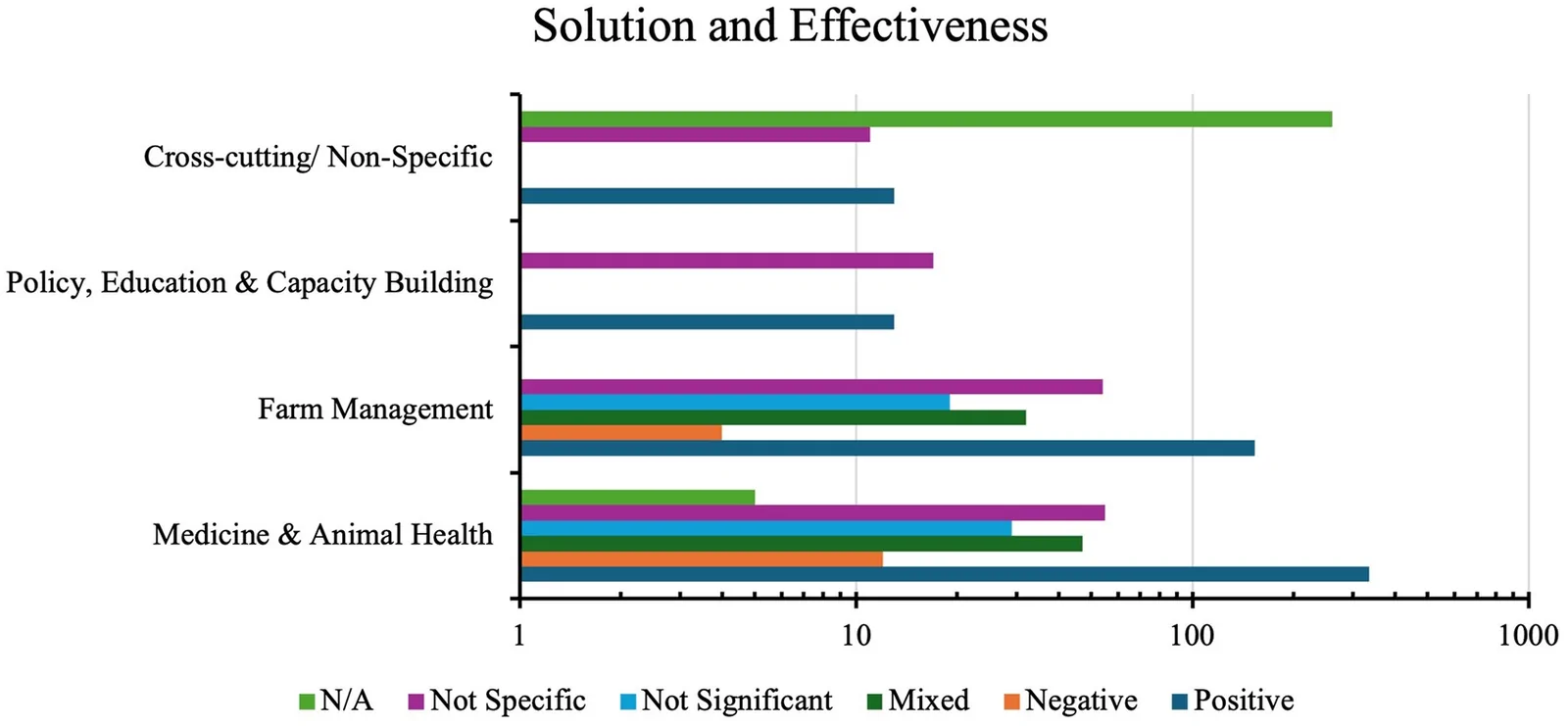
Solution and effectiveness.

### Interactive maps

Three interactive maps were generated to help with the visualization of the results. The maps were stored in the Texas Data Repository (https://doi.org/10.18738/T8/Z0JQAO). Map l focuses on threat categories, production phase and threat type. The size of the bubbles represents the number of studies. The color of the bubbles represents threat type, and the location of the bubbles represent the threat categories under a specific production phase. Figure 15 presents the screenshot of the interactive map 1. The evidence gap map clearly shows that biological threats are represented by the largest bubbles, indicating that they are the most extensively studied threat category. These threats are primarily concentrated in the veterinary services, grass-finishing, feedlot, and cow-calf production phases. Map 2 includes specific disease, production phase, and threat impact. The columns represent the production phase. The rows represent specific diseases. The size of the bubbles represents the number of studies. The color of the bubbles represents the threat impact. The location of the bubble represents the intersection of the specific disease and specific production phase. Map 3 includes threat categories, solution, and effectiveness. The columns represent the solution. The rows represent the threat category. The size of the bubbles represents the number of studies. The color of the bubbles represents the effectiveness. The location of the bubble represents the intersection of threat categories and solutions. To help the reader understand how to use the interactive maps, we provided a user guide handbook as supplementary material B, available as supplementary data at *Translational Animal Science* online. It is not a reflection of the findings of this paper, rather, serves as a manual of how to use the interactive maps effectively. Additional details are provided in Figs S1–S9, available as supplementary data at *Translational Animal Science* online.

**Figure 15:**
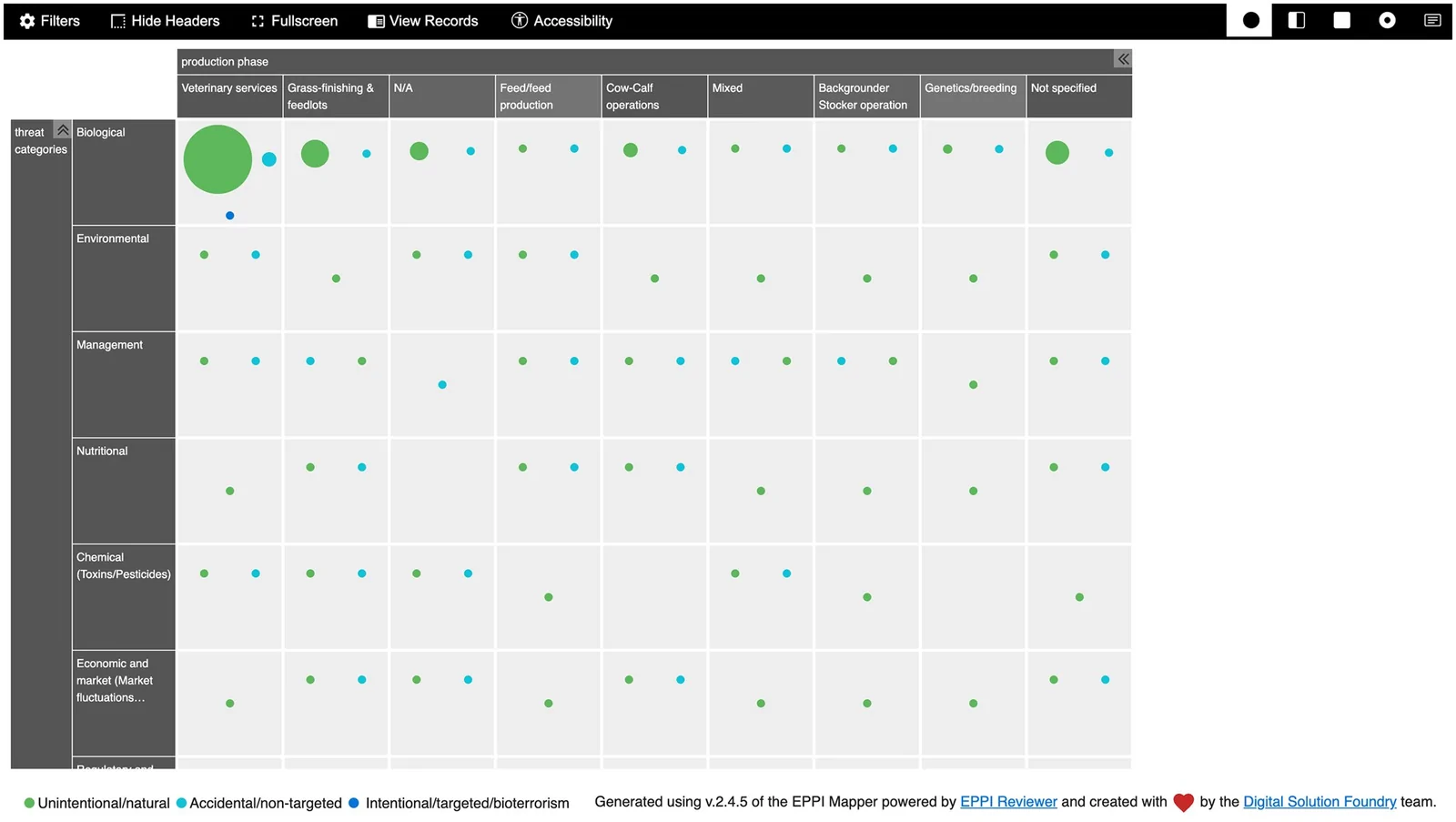
Screenshot of interactive map 1.

## Discussions

### Research trends in U.S. beef production

A broad review of the literature suggests that research in beef production has expanded steadily over time, reflecting growing scholarly attention to challenges and opportunities within the beef industry. The steady increase in research from 2010 to 2021 reflects growing recognition of vulnerabilities in beef production in scientific literature. The more pronounced surge after 2015 may relate to heightened attention toward emerging zoonotic diseases, antimicrobial resistance, and environmental stressors affecting livestock systems (Bernstein et al. 2022; Kappes et al. 2023). The sharp rise in 2020 to 2021 aligns with the COVID-19 pandemic, as Bernstein et al. (2022) claim that coronavirus most likely originated from animal sources, which exposed critical weaknesses in global and national food supply chains, prompting new studies on biosecurity, workforce resilience, and logistics continuity. Moreover, national security concerns, particularly regarding agroterrorism, may have reinforced this research trend, as the U.S. agriculture sector is a potential target of agroterrorism (Ashlock et al. 2012; Adebola 2021).

#### Methodological emphasis and evidence gaps

The dominance of lab and field testing/pilot studies indicates that much of the research emphasizes biological or environmental threats, often within controlled contexts. This pattern suggests that the evidence base is technically strong but context-specific, more investigating needs to be done before translating those lab results to farm-level practice or national-scale decision-making. The limited share of policy and invention studies highlights a narrow methodological scope, research is heavily weighted toward empirical/technical testing rather than systemic or governance-level risk evaluation. The small proportion of modeling/theoretical studies provide potential for predictive or scenario-based evaluation of interventions, but their modest number limits evidence synthesis across risk types. Together, these patterns indicate that while empirical data are strong, there’s less balance in theoretical and policy-oriented perspectives necessary for holistic supply chain risk assessment. These findings suggest that while the beef production literature is extensive and strongly oriented toward applied research, there remain opportunities for diverse study types.

A key gap of the evidence base is the sparse representation of policy, education, regulatory, and adoption-oriented research, which limits conclusions about how technical interventions are implemented, coordinated, and sustained in commercial beef production. This gap is consequential because producer adoption is shaped by practical, economic, social, and operation-specific conditions rather than by knowledge alone; Pudenz et al. (2021), for example, documented low adoption of several pre-outbreak practices associated with the Secure Beef Supply Plan (2025), while Buchan et al. (2023) identified interacting economic, practical, and social influences on disease-control adoption. Future research should therefore integrate technical evaluations with implementation and economic analyses, including feasibility, institutional capacity, stakeholder costs and benefits, and the roles of veterinarians, Extension professionals, producer organizations, industry groups, and animal-health agencies in supporting operation-specific adoption and coordinated preparedness (Charlier et al. 2022; Kappes et al. 2023).

#### Scale mismatch between study design and threat framing

The findings of study sites and threats impact suggests that researchers tend to use small scale design to study big problems. The study site refers to where the research was conducted or where data were collected. The results for the study site reveals that research on risks within the beef industry predominantly focuses on localized contexts, with comparatively fewer studies conducted at broader regional, national, or global scale. Threat impacts refers to At what scale the threat is believed to affect the beef industry. The results from threat impact indicates that most studies assess threats impact primarily at national and global scales, less studies focus on threats that only affect at the state or individual level. The higher research interest in big problems that can potentially impact the industry at national level or global level reflect that scholars and institutions may prioritize addressing systemic and cross-border risks, likely due to their wider societal, economic, and policy implications. In practice, researchers frequently use localized data and case-specific investigations to understand broader challenges or issues affecting the whole beef industry. The use of small scale or localized research design might be related to limited resources, available funding or reflects the localized nature of many production risks and management practices. However, it is still debatable of the generalization of the small scale design–relying heavily on local evidence to inform larger-scale risks decision making. While localized studies provide valuable insights, additional regional, national, and cross-national research may be needed to fully capture the complexity and variability of threats globally and nationally. Future research is encouraged to investigate broader patterns and system-level issues that transcend individual locations. Greater attention to larger-scale analyses could support more comprehensive understanding of risks and resilience within the beef production sector. In addition, the relatively limited attention associated with state and individual-level threats indicates a potential gap in localized and person-centered objectives and scope, which are also critical for understanding variability in vulnerability, adaptive capacity, and intervention effectiveness. A more balanced research portfolio could therefore enhance multilevel threat preparedness and response strategies by integrating macro-level trends with context-specific insights.

### Biological threat most dominant

#### Distribution of biological threat research across phases

The fact that biological hazards accounted for three-quarters of the retained literature (793 of 1,064 records, 74.5%) is less informative than within the production chain, where the core attention of our study settled. Nearly half of the biological records (368, 46.4%) were assigned to veterinary services, and a further 105 (13.2%) to grass-finishing and feedlots, while cow-calf operations attracted 56 records (7.1%), backgrounding and stocker operations 16 (2.0%), and feed production 15 (1.9%). This distribution does not follow the normal distribution of cattle. Most U.S. beef cows are held in small, dispersed, pasture-based herds: the 2022 Census of Agriculture recorded that 55.0% of farms with beef cows carried fewer than 20 head, and the average beef cow herd was approximately 47 head (USDA-NASS 2022; USDA-ERS 2024). For most small and medium operations, the enterprise is a supplementary rather than a primary source of income (USDA-APHIS 2017). Feedlots, by contrast, concentrate large numbers of animals under continuous observation, with individual identification, treatment records, and routine veterinary contact. The phase distribution we observed, therefore, tracks the availability of research infrastructure rather than the location of risk. The consequence is that the segments in which an introduced or emerging agent would most plausibly circulate before detection, namely the cow-calf and stocker phases, are the segments for which the evidence base is thinnest. Chen et al. (2022) reached a similar conclusion from a narrower starting point, reporting that most BRD prevention research applicable to cow-calf systems had in fact been generated in feedlot settings and transferred by inference.

#### Emphasis on BRD relative to other conditions

The concentration on a small number of conditions is easier to uphold. Bovine respiratory disease (BRD) alone accounted for 233 records, 29.4% of the biological set, and, at first reading, this appears disproportionate compared with the 51 conditions that appeared only once. Set against the burden, however, the emphasis is broadly proportionate. BRD has long been reported to account for roughly three-quarters of feedlot morbidity and between half and seventy percent of feedlot mortality (Edwards 1996; Galyean et al. 1999; Loneragan et al. 2001), with direct annual losses to the U.S. industry estimated at $800 to $900 million (Chirase and Greene 2001) and per-case treatment costs recorded by NAHMS rising from $12.59 in 1999 to $23.60 in 2011 (USDA-APHIS 2013). A research portfolio that devotes about three of every ten biological studies to the single most costly endemic condition in the sector is behaving rationally. The difficulty identified by this map is not that BRD received too much attention, but that volume of attention did not translate into resolution.

#### Declining etiological resolution and lack of standardization

This is a profound finding of the present analysis. Only 385 biological records (48.5%) identified a causal agent at all, and the deficit was concentrated in precisely the condition that attracted the most study: 158 of the 233 BRD records (67.8%) named no pathogen, and among the 75 that did, Mannheimia haemolytica accounted for 41, while viral agents appeared in fewer than twenty records combined. Some aggregation is justifiable, since BRD is a complex in which viral infection (bovine respiratory syncytial virus, bovine herpesvirus 1, bovine viral diarrhea virus, parainfluenza-3) predisposes animals to opportunistic colonization by M. haemolytica, Pasteurella multocida, Histophilus somni, and Mycoplasma bovis (Neibergs et al. 2014). Yet the pattern we observed is consistent with a documented failure of standardization rather than with deliberate methodological choice. Ferraro et al. (2021), reviewing 23 studies of BRD case definition, found 22 distinct combinations of reporting criteria, only two studies sharing a definition, and no study reporting any measure of agreement. Apley (2014) had already framed the underlying difficulty in comparable terms, identifying the evaluation of preventive and therapeutic interventions as compromised whenever production system data are the sole basis for analysis, alongside related problems of predicting morbidity within groups and diagnosing individual cases accurately. Our data extends that observation from the clinical to the etiological level, and in an unexpected direction: agent specification declined over the study period, from 54.9% of biological records in 2010 to 2016 to 43.4% in 2017 to 2024, and within the BRD subset from 36.3% to 27.5%. That decline spans the implementation of FDA Guidance for Industry #213 and the revised Veterinary Feed Directive on 1 January 2017, which withdrew medically important antimicrobials from production use and placed in-feed administration under veterinary oversight (FDA 2015). Stewardship decisions of this kind depend on knowing which organism is being treated. Part of the shift plausibly reflects a legitimate movement toward production-level, precision-livestock, and genomic study designs in which the individual agent is not the unit of interest, and we cannot separate the two effects with mapping data. Even allowing for that, a literature in which agent-level resolution is falling while regulatory demand for it is rising is not well positioned to support vaccine matching, diagnostic development, or stewardship policy.

#### Regulatory visibility and producer awareness

The mismatch between this research profile and producer knowledge is best read through the same lens. Producers surveyed by USDA reported greatest familiarity with brucellosis, foot-and-mouth disease, bovine viral diarrhea, bovine spongiform encephalopathy, and trichomoniasis, conditions that together accounted for 107 biological records (13.5%), fewer than half the number devoted to BRD. It would be a mistake to read this as producer inattention, because those five conditions share a feature that BRD does not. Brucellosis and bovine tuberculosis are administered through cooperative State-Federal eradication programmes that impose testing, identification, movement, and herd-certification requirements on producers directly (USDA-APHIS 2024a, 2024b). Trichomoniasis is a reportable disease in Texas and other states, and bull testing is required before a change of ownership or commingling (TAHC 2024). BSE carries surveillance and feed-ban obligations. FMD is a foreign animal disease, and it is governed by a national response plan. Each therefore reaches the producer through a regulatory channel, in the form of a test chart, a movement permit, or a certificate, irrespective of how much or how little is published about it. BRD reaches the producer through no such channel; it is managed at the operation and absorbed into the cost of gain. Producer familiarity in this sector appears to track regulatory contact rather than either research volume or economic burden. That reframes the extension problem materially. The gap is not that producers have failed to absorb the literature, but that the literature has no delivery mechanism comparable to the one that carries programme diseases, and extension planning that assumes publication volume will translate into producer awareness misreads how the information actually travels.

#### Anticipatory research and the FMD exception

The foot-and-mouth disease records warrant separate comment, because they are the one part of the biological group that anticipates rather than responds. FMD has not been detected in U.S. livestock since 1929 (AVMA 2024), so these 45 records documents no domestic burden; they are preparedness work, and their composition reflects it, with 11 of 45 classified as modeling or theoretical studies and 27 assessed as global in scope, both proportions well above the biological average. That posture is warranted. Modeled costs of a U.S. incursion range from $6 to $14 billion for an outbreak confined to California (USDA-APHIS 2020), and Government Accountability Office (GAO 2019) found USDA preparedness improvements were incomplete at the time of review. The question this map raises is why anticipatory research is confined almost entirely to one foreign animal disease, when the same reasoning, that of low probability, high consequence, and no domestic case series from which to learn, applies to a range of other potential introductions. Taken together, these findings indicate that biological threats dominate this literature in volume without delivering proportionate depth. The concentration is justified where burden justifies it, but coverage is shallow at the level of causal agent, thin upstream of the feedlot, and, outside of FMD, almost wholly reactive.

### Patterns and implications of reported mitigation strategies

#### Distribution of mitigation strategies in the literature

The distribution of reported mitigation strategies broadly reflected the threat profile identified in this mapping review. Figure 16 demonstrates the screenshot of interactive map 3. Vaccination and disease control were the most frequently reported strategies, followed by diagnostic and surveillance programs, and environmental and management practices. Together, these three categories accounted for more than half of the mapped evidence. This concentration was consistent with biological threats accounting for the majority of the included studies and with bovine respiratory disease being the most frequently reported specific disease. For instance, Fazzio et al. (2015) investigates the treatment to reduce BRD in grass-finishing & feedlot phase. The specific treatment is metaphylactic treatment with oxytetracycline (OTC) in the labeled and double doses, which yield a positive effect. Indicated by the interactive map 3, there is a notable lack of research on nutrition and supplementation, therapeutic approaches, policy and education initiatives, genetic and breeding solutions, and regulatory and compliance. Specifically, policy and education initiatives and regulatory/compliance approaches accounted for only a small proportion. That being reported, there is heavy weight on technical and animal-health intervention separate from policy, education, and regulatory implications.

**Figure 16:**
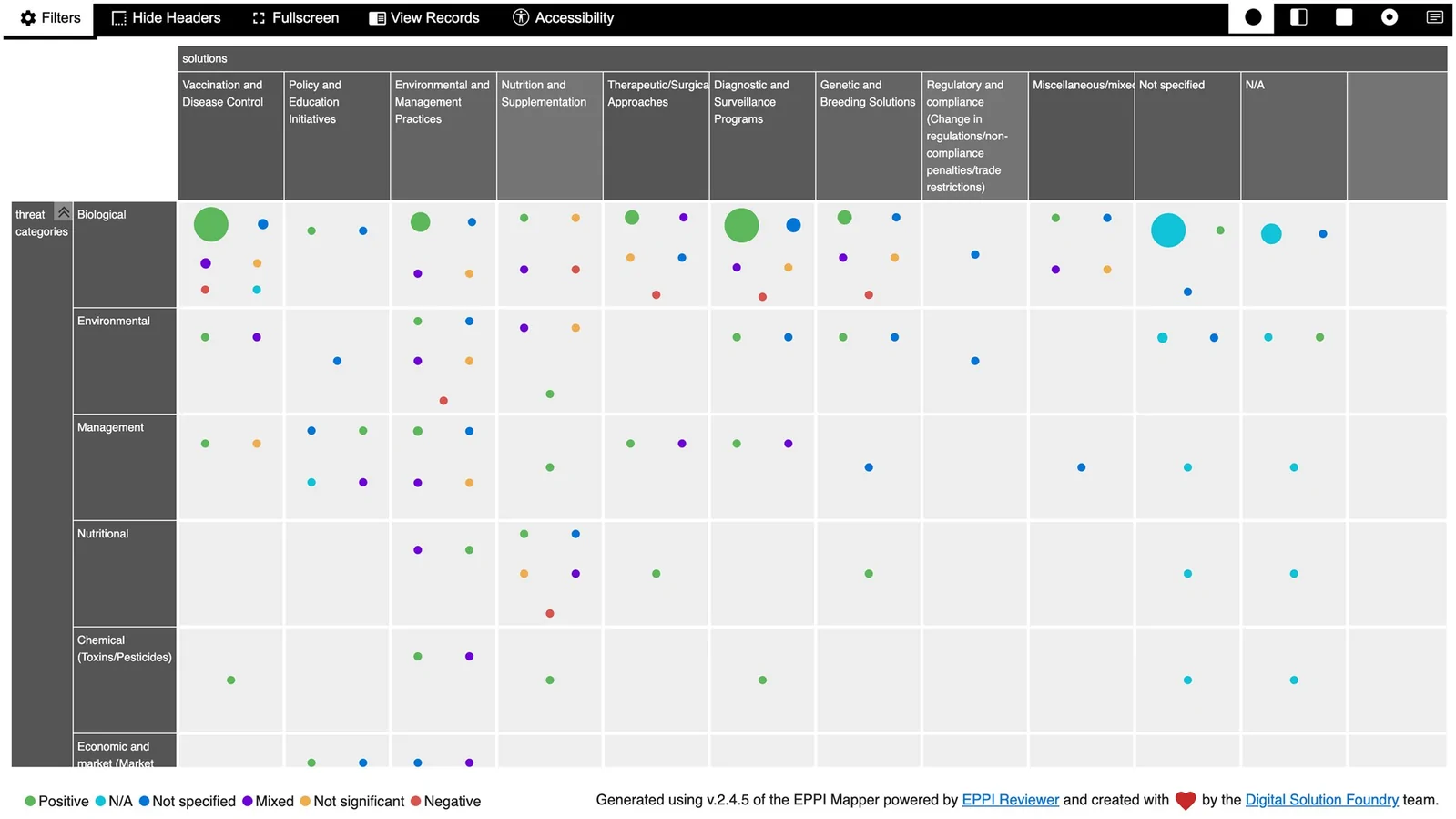
Screenshot of interactive map 3.

#### Context-dependent effects of vaccination and surveillance

The mapped evidence and previous syntheses suggest broad convergence around prevention, early detection, and production management as complementary components of mitigation. However, evidence for specific interventions remains heterogeneous across cattle populations and production conditions. Vaccination provides a clear example. McAllister et al. (2024) documented substantial variation in vaccination protocols and study populations. They reported antibody, health, and performance outcomes, whereas O’Connor et al. (2019) found limited compelling evidence that vaccination administered at or near feedlot arrival consistently reduced bovine respiratory disease diagnoses. These findings do not negate the role of vaccination. Rather, they indicate that its observed effects depend on cattle risk status, prior vaccination and management, timing of administration, pathogen exposure, and the outcome assessed. Vaccination is therefore best interpreted as one component of an operation-specific herd-health program rather than as a uniformly effective stand-alone strategy.

Diagnostic and surveillance programs were the second most frequently reported strategy, demonstrating substantial attention to the early recognition of disease and other production threats. Their practical value, however, depends on the accuracy, timeliness, and intended use of the diagnostic process. Timsit et al. (2016) found that clinical illness identified through routine observation had relatively low sensitivity but higher specificity for bovine respiratory disease in feedlot cattle, with considerable variation among studies. Surveillance must be a connected process involving observation, case definitions, diagnoses, and timely communication.

#### Environmental and management interventions and outcome reporting

Heterogeneity is also evident in environmental and management interventions. Environmental and management responses are similarly context dependent because their appropriateness is shaped by interactions among climatic hazards, exposure, production-system vulnerability, and adaptive capacity (Godde et al. 2021). Collectively, these findings support context-specific combinations of mitigation strategies rather than a one-size-fits-all approach.

Reported mitigation outcomes should be interpreted cautiously. Across the full corpus, 514 studies (48.3%) were coded as reporting positive outcomes, 81 (7.6%) as reporting mixed outcomes, 49 (4.6%) as reporting nonsignificant outcomes, and 16 (1.5%) as reporting negative outcomes. The remaining 404 studies (38.0%) were identified as either “not specified” or “not applicable” regarding mitigation outcomes. Although positive outcomes were the most frequently reported category, these proportions should not be interpreted as a pooled estimate of effectiveness. The included studies varied in research design, production system, intervention, comparator, outcome definition, and follow-up period; the mapping review was designed to classify the evidence rather than to compare intervention effects statistically. This caution is reinforced by the composition of the evidence base: laboratory, field-testing, and pilot studies accounted for 59.7% of the included studies, whereas intervention, diffusion, and adoption studies accounted for 12.7%, and policy studies accounted for only 0.4%. Accordingly, the map provides stronger evidence about where mitigation research is concentrated and how outcomes have been reported than about the comparative effectiveness or generalizability of individual strategies.

As a mapping review, this study was not designed to identify a single best mitigation strategy. Instead, the findings support interpreting mitigation approaches as complementary and context-dependent, with their relevance varying according to the threat, cattle population, production phase, and operational setting. The map’s principal contribution is to show where mitigation evidence is concentrated, where reported outcomes vary, and which dimensions of threat management remain comparatively underrepresented.

### Limitations and future directions

This mapping review is, to our knowledge, the first to synthesize the available evidence from published literature over time, address critical threats to the beef production, and bridge existing gaps in the research. We employed a rigorous and replicable methodological approach, ensuring transparency throughout the process. However, this systematic mapping study has several limitations.

First, the study primarily focused on threats occurring during the beef production phase. Future research could extend the scope beyond production to other stages of the beef supply chain, including processing, distribution, and marketing/consumption. Second, the definition and inclusion criteria for “cross-border” threats were relatively broad and may have introduced inconsistencies in study selection. Future studies should provide a clearer operational definition and apply more stringent inclusion criteria to improve consistency and transparency. Third, the review was limited to publications from 2010 to 2024. As new risks emerge–for example, screwworms threat to the Texas beef industry–future research can expand the timeframe to capture the most current evidence, and may also benefit from narrower, threat-specific syntheses that allow more in-depth analyses of particular issues affecting the beef industry.

Fourth, this study did not specifically examine the environmental impacts and consequences of threats to beef production. Future research could explore the environmental dimensions of these impacts, including their implications for sustainability and ecosystem health. Additionally, although the use of antibiotics and the emergence of antimicrobial resistance represent significant challenges for the beef industry and pose important public health concerns, this study focuses more on animal-related issues instead of human health-related issues, which were not a major focus of the present study. Future research could investigate topics such as antimicrobial resistance, antibiotic use in beef production, and foodborne illnesses in greater depth. Finally, the review included only English-language publications. As a result, relevant studies published in other languages were excluded. Future reviews should consider incorporating non-English literature to provide a more comprehensive and globally representative understanding of threats to the beef supply chain. Due to constraints in human resources, we only searched one database and grey literature produced from several research organizations, and included proceedings of a couple conferences. Future research is encouraged to explore multiple databases.

## Conclusions

This study conducted a comprehensive mapping review of potential threats to the U.S. beef supply chain in the production phase. It identified research trends and systematically categorized risks, including threat categories, specific diseases and pathogens, their impacts, and solutions/strategies. A search based on CAB Abstracts and major research organizations’ grey literature and major conference proceedings included 1064 studies for review. We found steadily increasing scholarly attention from 2010 to 2024. Research is highly focused on empirical applied research, while there is a lack of other types, such as modeling, theoretical, or policy papers. Researchers tend to use small-scale designs at the local level to study issues and challenges that pose global and national impact. Biological threat was the most dominant, with BRD being the most studied area. The solutions are predominantly focused on vaccination and disease control, diagnostic and surveillance programs, as well as environmental and management practices, while indicating a notable lack of research on nutrition and supplementation, therapeutic approaches, policy and education initiatives, genetic and breeding solutions, and regulatory and compliance.

## Supplementary Material

txag143_Supplementary_Data

## List of abbreviations

U.S.: United States

## References

[txag143-B1] Abramovitz M. 1956. Resource and output trends in the United States since 1870. In: Resource and output trends in the United States since 1870. NBER, New York, NY. p. 1–23. https://www.nber.org/books-and-chapters/resource-and-output-trends-united-states-1870/resource-and-output-trends-united-states-1870

[txag143-B2] Adebola O. 2021. Analyzing the threat, vulnerability, and consequences of agroterrorism. In: Kosal ME, editors. Proliferation of weapons- and dual-use technologies. Advanced sciences and technologies for security applications. Springer, Cham. 10.1007/978-3-030-73655-2_14

[txag143-B3] Aherin D. G. , Weaber R. L., Pendell D. L., Heier Stamm J. L., Larson R. L. 2023. Stochastic, individual animal systems simulation model of beef cow–calf production: development and validation. Transl. Anim. Sci. 7:txac155. 10.1093/tas/txac155 PMC9930734 36816825

[txag143-B4] Albin R. C. 1998. The US beef cattle industry: an overview. https://www.cabidigitallibrary.org/doi/pdf/10.5555/20063209622

[txag143-B5] Apley M. 2014. The clinical syndrome of BRD: What it is and what it is not. Anim. Health Res. Rev. 15:135–137. 10.1017/S1466252314000152 25381807

[txag143-B6] Ashlock M. , Cartmell D., Leising J. 2012. Beef producers’ risk perceptions of an agroterrorism event occurring in Oklahoma. J. Appl. Commun. 96:65–73. 10.4148/1051-0834.1151

[txag143-B7] Ashlock M. , Leising J., Cartmell D. 2009. Agroterrorism and the implications of uncertainty reduction theory for agricultural communicators. J. Appl. Commun. 93:44–55. 10.4148/1051-0834.1200

[txag143-B8] AVMA. 2024. Foot and mouth disease preparedness. https://www.avma.org/resources-tools/avma-policies/foot-and-mouth-disease

[txag143-B9] Balagtas J. V. , Cooper J. 2021. The impact of COVID-19 on United States meat and livestock markets. Choices. 36:1–10.

[txag143-B10] Barrett J. R. et al 2021. Consumer perceptions of antimicrobial use in animal husbandry: a scoping review. PLoS One. 16:e0261010. 10.1371/journal.pone.0261010 PMC8654221 34879112

[txag143-B11] Bernstein A. S. et al 2022. The costs and benefits of primary prevention of zoonotic pandemics. Sci. Adv. 8:eabl4183. 10.1126/sciadv.abl4183 PMC8816336 35119921

[txag143-B12] Berry D. P. , Bermingham M. L., Good M., More S. J. 2011. Genetics of animal health and disease in cattle. Ir. Vet. J. 64:5. 10.1186/2046-0481-64-5 PMC3102331 21777492

[txag143-B13] Bina J. D. , Tonsor G. T., Schulz L. L., Hahn W. F. 2022. Regional and plant-size impacts of COVID-19 on beef processing. Food Policy. 108:102247. 10.1016/j.foodpol.2022.102247 PMC9757989 36569118

[txag143-B14] Buchan M. S. , Lhermie G., Mijar S., Pajor E., Orsel K. 2023. Individual drivers and barriers to adoption of disease control and welfare practices in dairy and beef cattle production: a scoping review. Front. Vet. Sci. 10:1104754. 10.3389/fvets.2023.1104754 PMC10357041 37483294

[txag143-B15] Cameron A. , McAllister T. A. 2016. Antimicrobial usage and resistance in beef production. J. Anim. Sci. Biotechnol. 7:68. 10.1186/s40104-016-0127-3 PMC5154118 27999667

[txag143-B16] Caswell J. L. et al 2018. Observational study design in veterinary pathology, part 1: study design. *Veterinary.* Pathology. 55:607–621. 10.1177/0300985818785705 30071806

[txag143-B17] Charlier J. et al 2022. Disease control tools to secure animal and public health in a densely populated world. Lancet Planet. Health. 6:e812–e824. 10.1016/S2542-5196(22)00147-4 36208644

[txag143-B18] Chen S. Y. , Negri Bernardino P., Fausak E., Van Noord M., Maier G. 2022. Scoping review on risk factors and methods for the prevention of bovine respiratory disease applicable to cow-calf operations. Animals. (Basel). 12:Article 334. 10.3390/ani12030334 PMC8833575 35158660

[txag143-B19] Chirase N. K. , Greene L. W. 2001. Dietary zinc and manganese sources administered from the fetal stage onwards affect immune response of transit stressed and virus infected offspring steer calves. Anim. Feed Sci. Technol. 93:217–228. 10.1016/S0377-8401(01)00277-2

[txag143-B20] Cowley C. 2022. Long-term pressures and prospects for the US cattle industry. Econ. Rev. 107:23–43. 10.18651/ER/v107n1Cowley

[txag143-B21] Crist S. , Mori J., Smith R. L. 2020. Flooding on beef and swine farms: a scoping review of effects in the Midwestern United States. Prev. Vet. Med. 184:105158. 10.1016/j.prevetmed.2020.105158 33045630

[txag143-B22] Cupp O. S. , Walker D. E., Hillison J. 2004. Agroterrorism in the U.S.: key security challenge for the 21st century. Biosecur. Bioterror. 2:97–105. 10.1089/153871304323146397 15225403

[txag143-B23] Di Girolamo N , Meursinge Reynders R. 2016. Deficiencies of effectiveness of intervention studies in veterinary medicine: a cross-sectional survey of ten leading veterinary and medical journals. PeerJ. 4:e1649. 10.7717/peerj.1649 PMC4734056 26835187

[txag143-B24] Done H. Y. , Venkatesan A. K., Halden R. U. 2015. Does the recent growth of aquaculture create antibiotic resistance threats different from those associated with land animal production in agriculture? AAPS J. 17:513–524. 10.1208/s12248-015-9722-z PMC4406955 25700799

[txag143-B25] Economic Research Service. 2024. Cattle and beef: sector at a glance. U.S. Department of Agriculture.https://www.ers.usda.gov/topics/animal-products/cattle-beef/sector-at-a-glance

[txag143-B26] Edwards A. 1996. Respiratory diseases of feedlot cattle in central USA. Bov. Pract. (Stillwater). 30:5–7. 10.21423/bovine-vol1996no30p5-7

[txag143-B27] Fazzio L. E. , Giuliodori M. J., Galvan W. R., Streitenberger N., Landoni M. F. 2015. A metaphylactic treatment with double dose oxytetracycline reduces the risk of bovine respiratory disease in feedlot calves. Rev. Vet. 26:89–92. https://revistas.unne.edu.ar/index.php/vet/article/view/213/183

[txag143-B28] FDA. 2015. Veterinary feed directive (Final rule). U.S. Food and Drug Administration (FDA), Silver Spring, MD.

[txag143-B29] Ferdousi T. , Gruenbacher D., Scoglio C. M. 2020. A permissioned distributed ledger for the US beef cattle supply chain. IEEE Access. 8:154833–154847. 10.1109/ACCESS.2020.3019000

[txag143-B30] Fernandez-Novo A. , Pérez-Garnelo S. S., Villagrá A., Pérez-Villalobos N., Astiz S. 2020. The effect of stress on reproduction and reproductive technologies in beef cattle—a review. Animals. 10:2096. 10.3390/ani10112096 PMC7697448 33187308

[txag143-B31] Ferraro S. et al 2021. Scoping review on clinical definition of bovine respiratory disease complex and related clinical signs in dairy cows. J. Dairy Sci. 104:7095–7108. 10.3168/jds.2020-19471 33741167

[txag143-B32] Fulton R. W. , Confer A. W. 2012. Laboratory test descriptions for bovine respiratory disease diagnosis and their strengths and weaknesses: gold standards for diagnosis, do they exist? Can. Vet. J. 53:754–761. PMC3377458 23277642

[txag143-B33] Gadberry M. S. et al 2019. Arkansas beef cattle industry: 2017 self-assessment. Appl. Anim. Sci. 35:357–370. 10.15232/aas.2019-01859

[txag143-B34] Galyean M. L. , Perino L. J., Duff G. C. 1999. Interaction of cattle health/immunity and nutrition. J. Anim. Sci. 77:1120–1134. 10.2527/1999.7751120x 10340578

[txag143-B35] GAO. 2019. Foot-and-mouth disease: USDA’s efforts to prepare for a potential outbreak could be strengthened (GAO-19-103). U.S. Government Accountability Office, Washington, DC.

[txag143-B36] GAP Initiative. 2018. USA: a powerful legacy, with room to grow | Virginia Tech CALS Global. https://globalagriculturalproductivity.org/sustainable-food-and-agriculture-systems-are-built-on-productivity/regional-productivity-growth-progress-and-challenges/usa-a-productivity-powerhouse-loses-some-steam/

[txag143-B37] Gill K. M. 2015. Agroterrorism: the risks to the United States food supply and national security. U.S. Army Med. Dep. J. 9–15. https://go.gale.com/ps/i.do?asid=20ca4c59&id=GALE%7CA406709594&it=r&p=AONE&sid=AONE&u=googlescholar&v=2.1 25651140

[txag143-B38] Godde C. M. , Mason-D’Croz D., Mayberry D. E., Thornton P. K., Herrero M. 2021. Impacts of climate change on the livestock food supply chain; a review of the evidence. Glob. Food Sec. 28:100488. 10.1016/j.gfs.2020.100488 PMC7938222 33738188

[txag143-B39] Gollop F. M. , Swinand G. P. 1998. From total factor to total resource productivity: an application to agriculture. Am. J. Agri. Econ. 80:577–583. 10.2307/1244562

[txag143-B40] Greig J. et al 2015. A scoping review of the role of wildlife in the transmission of bacterial pathogens and antimicrobial resistance to the food chain. Zoonoses Public Health. 62:269–284. 10.1111/zph.12147 25175882

[txag143-B41] Hatfield J. L. , Prueger J. H. 2015. Challenge for future agriculture. In: Crop wild relatives and climate change. John Wiley & Sons, Ltd, Chichester, UK. p. 24–43. 10.1002/9781118854396

[txag143-B42] Havstad K. M. et al 2018. Vulnerabilities of Southwestern U.S. Rangeland-based animal agriculture to climate change. Clim. Change. 148:371–386. 10.1007/s10584-016-1834-7

[txag143-B43] Hayes D. , Jacobs K., Schulz L., Crespi J. 2023. Resilience of U.S. Cattle and Beef Sectors: lessons from COVID-19. J. Agric. Food Ind. Organ. 21:53–67. 10.1515/jafio-2022-0021

[txag143-B44] Hristov A. N. et al 2018. Climate change effects on livestock in the Northeast US and strategies for adaptation. Clim. Change. 146:33–45. 10.1007/s10584-017-2023-z

[txag143-B45] Hubbart J. A. et al 2023. Challenges in sustainable beef cattle production: a subset of needed advancements. Challenges. (Basel). 14:14. 10.3390/challe14010014

[txag143-B46] James K. L. , Randall N. P., Haddaway N. R. 2016. A methodology for systematic mapping in environmental sciences. Environ. Evid. 5:1–13.

[txag143-B47] Jones B. A. et al 2013. Zoonosis emergence linked to agricultural intensification and environmental change. Proc. Natl. Acad. Sci. USA. 110:8399–8404. 10.1073/pnas.1208059110 PMC3666729 23671097

[txag143-B48] Jorquera-Chavez M. et al 2020. Remotely sensed imagery for early detection of respiratory disease in pigs: a pilot study. Animals. (Basel). 10:451. 10.3390/ani10030451 PMC7142473 32182745

[txag143-B49] Kappes A. et al 2023. Livestock health and disease economics: a scoping review of selected literature. Front. Vet. Sci. 10:1168649. 10.3389/fvets.2023.1168649 PMC10546065 37795016

[txag143-B50] Knox P. , Fuhrmann C., Konrad C. 2014. Challenges and opportunities for southeast agriculture in a changing climate: perspectives from state climatologists. SGO. 54:118–136.

[txag143-B51] Landers T. F. , Cohen B., Wittum T. E., Larson E. L. 2012. A review of antibiotic use in food animals: perspective, policy, and potential. Public Health Rep. 127:4–22. 10.1177/003335491212700103 PMC3234384 22298919

[txag143-B52] Lhermie G. et al 2019. Antimicrobial policies in United States beef production: choosing the right instruments to reduce antimicrobial use and resistance under structural and market constraints. Front. Vet. Sci. 6:245. 10.3389/fvets.2019.00245 PMC6658893 31380404

[txag143-B53] Lhermie G. et al 2020. Economic effects of policy options restricting antimicrobial use for high risk cattle placed in U.S. feedlots. PLoS One. 15:e0239135. 10.1371/journal.pone.0239135 PMC7491722 32931522

[txag143-B54] Li S. , Subbiah J., Dvorak B. 2019. Environmental and occupational impacts from U.S. beef slaughtering are of same magnitude of beef foodborne illnesses on human health. Environ. Int. 129:507–516. 10.1016/j.envint.2019.05.051 31170666

[txag143-B55] Loneragan G. H. , Dargatz D. A., Morley P. S., Smith M. A. 2001. Trends in mortality ratios among cattle in US feedlots. J. Am. Vet. Med. Assoc. 219:1122–1127. 10.2460/javma.2001.219.1122 11700712

[txag143-B56] Lowe M. , Gereffi G. 2009. A value chain analysis of the US beef and dairy industries. In: Center on globalization, governance & competitiveness. Duke University, Durham, NC. p. 1–55.

[txag143-B57] Ma Z. , Lee S., Jeong K. C. 2019. Mitigating antibiotic resistance at the livestock-environment interface: a review. J. Microbiol. Biotechnol. 29:1683–1692. 10.4014/jmb.1909.09030 31693837

[txag143-B58] Mandel R. , Bracke M. B. M., Nicol C. J., Webster J. A., Gygax L. 2022. Dairy vs beef production–expert views on welfare of cattle in common food production systems. Animal. 16:100622. 10.1016/j.animal.2022.100622 36109300

[txag143-B59] McAllister H. R. et al 2024. A systematic review on the impact of vaccination for respiratory disease on antibody titer responses, health, and performance in beef and dairy cattle. Vet. Sci. 11:599. 10.3390/vetsci11120599 PMC11680273 39728939

[txag143-B60] Miranowski J. A. , Scanes C. G. 2008. Perspectives in world food and agriculture 2004. John Wiley & Sons, Ames, Iowa. 10.1002/9780470290187

[txag143-B61] Monke J. 2006. Agroterrorism: threats and preparedness. https://nationalaglawcenter.org/wp-content/uploads/assets/crs/RL32521.pdf

[txag143-B62] Montes de Oca Munguia O. , Pannell D. J., Llewellyn R. 2021. Understanding the adoption of innovations in agriculture: a review of selected conceptual models. Agronomy. 11:139. 10.3390/agronomy11010139

[txag143-B63] Morgavi D. P. , Riley R. T. 2007. An historical overview of field disease outbreaks known or suspected to be caused by consumption of feeds contaminated with Fusarium toxins. Anim. Feed Sci. Technol. 137:201–212. 10.1016/j.anifeedsci.2007.06.002

[txag143-B64] NASS. 2022. Census of Agriculture. U.S. Department of Agriculture. https://www.nass.usda.gov/Publications/AgCensus/2022/

[txag143-B65] Neibergs H. L. et al 2014. Susceptibility loci revealed for bovine respiratory disease complex in pre-weaned Holstein calves. BMC Genomics. 15:1164. Article 1164. 10.1186/1471-2164-15-1164 PMC4445561 25534905

[txag143-B66] O’Connor A. M. et al 2019. A systematic review and network meta-analysis of bacterial and viral vaccines, administered at or near arrival at the feedlot, for control of bovine respiratory disease in beef cattle. Anim. Health Res. Rev. 20:143–162. 10.1017/S1466252319000288 32081122

[txag143-B67] O’Neill K. 2005. U.S. beef industry faces new policies and testing for mad cow disease. Calif. Agric. J. UC Agric. Nat. Resour. 59:203–211. 10.3733/ca.v059n04p203

[txag143-B68] Peel D. S. et al 2020. Economic damages to the US beef cattle industry due to COVID-19. Oklahoma Cooperative Extension Service. https://shareok.org/bitstream/handle/11244/333064/oksa_CR-0630_2020-04.pdf?sequence=1

[txag143-B69] Penakalapati G. et al 2017. Exposure to animal feces and human health: a systematic review and proposed research priorities. Environ. Sci. Technol. 51:11537–11552. 10.1021/acs.est.7b02811 PMC5647569 28926696

[txag143-B70] Peterson H. H. et al 2023. Impacts of COVID-19 on US agri-food supply chain businesses: regional survey results. PLoS One. 18:e0281930. 10.1371/journal.pone.0281930 PMC9946254 36812219

[txag143-B71] Plastina A , Lence S. H. 2018. A parametric estimation of total factor productivity and its components in U.S. Agriculture. Am. J. Agri. Econ. 100:1091–1119. 10.1093/ajae/aay010

[txag143-B72] Pudenz C. C. , Mitchell J. L., Schulz L. L., Tonsor G. T. 2021. U. S. Cattle producer adoption of secure beef supply plan enhanced biosecurity practices and foot-and-mouth disease preparedness. Front. Vet. Sci. 8:660857. 10.3389/fvets.2021.660857 PMC8377467 34422939

[txag143-B73] Ramsey A. F. , Goodwin B. K., Hahn W. F., Holt M. T. 2021. Impacts of COVID‐19 and price transmission in U.S. meat markets. Agric. Econ. 52:441–458. 10.1111/agec.12628 PMC8206949 34149130

[txag143-B74] Richter B. D. et al 2020. Water scarcity and fish imperilment driven by beef production. Nat. Sustain. 3:319–328. 10.1038/s41893-020-0483-z

[txag143-B75] Riley K. , Cartmell D., Naile T. 2012. Kansas beef feedlot managers’ trusted sources of information concerning an agroterrorism event. J. Appl. Commun. 96:38–49. 10.4148/1051-0834.1152

[txag143-B76] Schaible G. D. , Aillery M. P. 2017. Chapter 2.1.1—challenges for US irrigated agriculture in the face of emerging demands and climate change. In: Ziolkowska J. R., Peterson J. M., editors. Competition for water resources. Elsevier, Amsterdam, Netherlands. p. 44–79. 10.1016/B978-0-12-803237-4.00004-5

[txag143-B77] Schaible G. , Aillery M. 2012. Water conservation in irrigated agriculture: trends and challenges in the face of emerging demands (SSRN Scholarly Paper 2186555). 10.2139/ssrn.2186555

[txag143-B78] Secure Beef Supply Plan. 2025. Guide to the secure beef supply plan. Center for Food Security and Public Health, Iowa State University, Ames, IO. https://www.securebeef.org/Assets/Guide-to-SBS-Plan.pdf

[txag143-B79] Smith R. A. 2013. The beef industry: tradition, technology, and threats. https://www.cabidigitallibrary.org/doi/pdf/10.5555/20133224962

[txag143-B80] Snilstveit B. , Vojtkova M., Bhavsar A., Gaarder M. 2013. Evidence gap maps-a tool for promoting evidence-informed policy and prioritizing future research. *World bank policy research working paper* (6725). SSRN. https://ssrn.com/abstract=2367606 10.1016/j.jclinepi.2016.05.015 27387966

[txag143-B81] Subak S. 1999. Global environmental costs of beef production. Ecol. Econ. 30:79–91. 10.1016/S0921-8009(98)00100-1

[txag143-B82] Sucena Afonso J. et al 2024. Strategies to reduce antimicrobials in livestock and aquaculture, and their impact under field conditions: a structured scoping literature review. J. Antimicrob. Chemother. 79:11–26. 10.1093/jac/dkad350 PMC10761277 37950886

[txag143-B83] TAHC. 2024. Cattle and bison health: trichomoniasis. https://www.tahc.texas.gov/animal_health/cattle/

[txag143-B84] Tedeschi L. O. , Menendez H. M. III. 2020. Mathematical modeling in animal production. In: Bazer F. W., Lamb G. C., Wu G., editors, Animal agriculture: sustainability, challenges and innovations. Academic Press, San Diego, CA. p. 431–453.

[txag143-B85] Teel P. D. , Hairgrove T. 2024. Transboundary tick and tick-borne pathogen threats to cattle. Vet. Clin. North Am. Food Anim. Pract. 40:305–316. 10.1016/j.cvfa.2024.01.006 38402041

[txag143-B86] Theuret D. P. , Trout Fryxell R. T. 2018. Beefing up biosecurity: survey of ticks (Acari: Ixodidae) currently threatening the Tennessee beef cattle industry, and a proposed monitoring strategy for invasive ticks. J. Med. Entomol. 55:1517–1526. 10.1093/jme/tjy131 30137368

[txag143-B87] Timsit E. , Dendukuri N., Schiller I., Buczinski S. 2016. Diagnostic accuracy of clinical illness for bovine respiratory disease (Brd) diagnosis in beef cattle placed in feedlots: a systematic literature review and hierarchical Bayesian latent-class meta-analysis. Prev. Vet. Med. 135:67–73. 10.1016/j.prevetmed.2016.11.006 27931931

[txag143-B88] USDA ERS. 2023. USDA ERS - sector at a glance. Economic Research Service U.S. DEPARTMENT OF AGRICULTURE. https://www.ers.usda.gov/topics/animal-products/cattle-beef/sector-at-a-glance/

[txag143-B89] USDA. 2020. A look at agricultural productivity growth in the United States, 1948–2017 | USDA. https://www.usda.gov/media/blog/2020/03/05/look-agricultural-productivity-growth-united-states-1948-2017

[txag143-B90] USDA-APHIS. 2013. Feedlot 2011 Part IV. USDA-APHIS, Fort Collins, CO.

[txag143-B91] USDA-APHIS. 2017. Beef 2017 cow-calf management practices in the United States. USDA-APHIS, Fort Collins, CO.

[txag143-B92] USDA-APHIS. 2020. Foot-and-mouth disease response plan: The red book. U.S. Department of Agriculture. https://www.aphis.usda.gov/livestock-poultry-disease/cattle/bovine-brucellosis

[txag143-B93] USDA-APHIS. 2024a. Bovine brucellosis.

[txag143-B94] USDA-APHIS. 2024b. Status of current eradication programs. U.S. Department of Agriculture. https://www.aphis.usda.gov/livestock-poultry-disease/status-eradication-programs

[txag143-B95] Veeck G. , Veeck A., Yu H. 2020. Challenges of agriculture and food systems issues in China and the United States. Geogr. Sustain. 1:109–117. 10.1016/j.geosus.2020.05.002

[txag143-B96] Wang S. L. , Heisey P., Schimmelpfennig D, Ball V. E. 2015. Agricultural productivity growth in the United States: measurement, trends, and drivers (SSRN Scholarly Paper 2692612). https://papers.ssrn.com/abstract=2692612

[txag143-B97] Weersink A. et al 2021. COVID-19 and the agri-food system in the United States and Canada. Agric. Syst. 188:103039. 10.1016/j.agsy.2020.103039 PMC7755574 33362333

[txag143-B98] White H. et al 2020. Guidance for producing a Campbell evidence and gap map. Campbell Syst. Rev. 16:e1125. 10.1002/cl2.1125 PMC8356343 37016607

[txag143-B99] Wiethoelter A. K. , Beltrán-Alcrudo D., Kock R., Mor S. M. 2015. Global trends in infectious diseases at the wildlife–livestock interface. Proc. Natl. Acad. Sci. USA. 112:9662–9667. 10.1073/pnas.1422741112 PMC4534210 26195733

[txag143-B100] Wortman S. E. , Lovell S. T. 2013. Environmental challenges threatening the growth of urban agriculture in the United States. J. Environ. Qual. 42:1283–1294. 10.2134/jeq2013.01.0031 24216408

[txag143-B101] Yang Q. et al 2019. Developing an agent-based model to simulate the beef cattle production and transportation in southwest Kansas. Phys. A. Stat. Mech. Appl. 526:120856. 10.1016/j.physa.2019.04.092

[txag143-B102] Zahniser S. , Taylor J. E., Hertz T., Charlton D., editors. 2018. Farm labor markets in the United States and Mexico Pose challenges for U.S. agriculture. 10.22004/ag.econ.281161

