# Supplementary material for: Systematic mapping of threats to U.S. beef production
Source: Transl Anim Sci. 2026 Sep 21;10:txag143. doi: 10.1093/tas/txag143 (PMC13677081; doi:10.1093/tas/txag143)
Supplement: Supplementary file 1 [file txag143_supplementary_data.zip › Supplementary Material A.docx]

Supplementary Material A

CAB Abstracts (Ovid) Search Strategies

# Search 1 - Feed (Input Supply Chain Phase) Search Strategy

1	exp beef cattle

2	(beef* adj1 cattle*).ab,ti.

3	cows

4	cow*.ab,ti.

5	exp calves/

6	calve*.ab,ti

7	calf*.ab,ti.

8	exp beef cows/

9	bulls/

10	bull*.ab,ti.

11	exp beef bulls/

12	exp heifers/

13	heifer*.ab,ti.

14	exp cattle/

15	cattle*.ab,ti.

16	veal/

17	veal*.ab,ti.

18	exp steers/

19	"steer*".ab,ti.

20	or/1-19

21	20 not ("dairy cattle*" or "dairy cow*" or "dairy calf" or "dairy calve*").ab,ti. [beef cattle terms]

22	exp cattle feeding/

23	exp calf feeding/

24	(cattle* adj2 feed*).ab,ti.

**25	or/22-24	[feed supply chain phase]**

26	exp agricultural land/

27	(agricultur* adj1 land).ab,ti.

28	exp overgrazing/

29	overgraz*.ab,ti.

30	exp climate change/

31	"climate change*".ab,ti.

32	"climate variabil*".ab,ti.

33	exp groundwater pollution/

34	(water adj1 pollution).ab,ti.

35	exp drought/

36	drought*.ab,ti.

37	exp soil degradation/

38	(soil adj1 degradat*).ab,ti.

39	exp soil management/

40	exp soil pollution/

41	exp fire/

42	fire*.ab,ti.

43	exp earthquakes/

44	earthquake*.ab,ti.

45	exp land degradation/

46	(land adj1 degradat*).ab,ti.

47	(rangeland adj1 degradat*).ab,ti.

48	exp natural disasters/

49	"natural disaster*".ab,ti.

50	"nuclear radiation* ".ab,ti.

51	(production adj1 facilit*).ab,ti.

52	exp factories/

53	exp crop quality/

54	"crop* quality".ab,ti.

55	exp crop yield/

56	"crop yield".ab,ti.

57	"crop load".ab,ti.

58	(yield adj2 reduct*).ab,ti.

59	exp feed contamination/

60	(feed* adj1 contaminat*).ab,ti.

61	(cross adj1 contaminat*).ab,ti.

62	(feed* adj1 short*).ab,ti.

63	(forage* adj1 short*).ab,ti.

64	(grass* adj1 short*).ab,ti.

65	exp pollutants/

66	pollutant*.ab,ti.

67	exp mycotoxins/

68	mycotoxin*.ab,ti.

69	exp lead/

70	exp dioxins/

71	dioxin*.ab,ti.

72	exp polychlorinated biphenyls/

73	"polychlorinated biphenyl*".ab,ti.

74	exp heavy metals/

75	"heavy metal*".ab,ti.

76	exp radionuclides/

77	radionuclide*.ab,ti.

78	exp feed rations/

79	(feed adj1 ration*).ab,ti.

80	exp portion size/

81	(portion* adj1 size*).ab,ti.

82	exp vitamins/

83	vitamin*.ab,ti.

84	exp feed supplements/

85	(feed adj1 supplement*).ab,ti.

86	exp feed additives/

87	(feed adj1 additive*).ab,ti.

88	exp nutrient intake/

89	(nutrient adj1 intake).ab,ti.

90	exp costs/

91	exp expenditure/

92	expenditure*.ab,ti.

93	expense*.ab,ti.

94	exp prices/

95	(price* adj2 feed*).ab,ti.

96	exp ethanol/

97	exp ethanol production/

98	ethanol*.ab,ti.

99	exp Salmonella/

100	exp Salmonella Dublin/

101	exp Salmonella Typhimurium/

102	salmonella*.ab,ti.

103	exp moulds/

104	mould*.ab,ti.

105	mold*.ab,ti.

106	exp fungi/

107	fungi*.ab,ti.

108	fungus.ab,ti.

109	exp Aspergillus flavus/

110	exp Aspergillus parasiticus/

111	"Aspergillus flavus".ab,ti.

112	Aspergillus parasiticus.ab,ti.

113	Aspergillus nomius.ab,ti.

114	exp sulfur/

115	sulfur*.ab,ti.

116	sulphur.ab,ti.

117	exp aflatoxins/

118	aflatoxin*.ab,ti.

119	exp spoilage/

120	spoilage*.ab,ti.

121	exp toxic substances/

122	"toxic substance*".ab,ti.

123	poison*.ab,ti.

124	exp ammonia/

125	ammonia.ab,ti.

**126	or/26-125	[feed threats]**

**127	21 and 25 and 126**

**128	limit 127 to yr="1960 - 2024"**

**Number of Records Retrieved: 10,325**

# Search 2 – Veterinary Services (Input Supply Chain Phase) Search Strategy

1	(recruit* adj2 veterinar*).ab,ti.

2	(retention adj2 veterinar*).ab,ti.

3	(retain* adj2 veterinar*).ab,ti.

4	exp parasites/

5	"deworm*".ab,ti.

6	exp brucellosis/

7	Brucellosis.ab,ti.

8	"Mycobacterium bovis".ab,ti.

9	exp cattle diseases/

10	exp calf diseases/

11	viral disease*.ab,ti.

12	exp mucosal disease/

13	mucosal disease*.ab,ti.

14	exp arboviruses/

15	arbovirus*.ab,ti.

16	exp Trypanosoma evansi/

17	Trypanosoma evansi.ab,ti.

18	exp leptospirosis/

19	leptospirosis.ab,ti.

20	exp bovine viral diarrhea virus 1/

21	"bovine viral diarrhea".ab,ti.

22	exp akabane virus/

23	akabane virus.ab,ti.

24	exp vibriosis/

25	vibriosis.ab,ti.

26	exp campylobacteriosis/

27	campylobacteriosis.ab,ti.

28	exp Enterobacteriaceae/

29	Enterobacteriaceae.ab,ti.

30	exp listeria monocytogenes/

31	listeria.ab,ti.

32	exp "foot and mouth disease"/

33	"foot and mouth disease*".ab,ti.

34	exp Salmonella/

35	Salmonella.ab,ti.

36	exp Campylobacter/

37	Campylobacter.ab,ti.

38	exp cysticercosis/

39	cysticercosis.ab,ti.

40	exp bovine herpesviruses/

41	(bovine adj1 herpesvirus*).ab,ti.

42	exp Bovine respiratory syncytial virus/

43	(Bovine adj1 respiratory).ab,ti.

44	exp bovine parainfluenza viruses/

45	"bovine parainfluenza".ab,ti.

46	exp infectious bovine keratoconjunctivitis/

47	(bovine adj1 keratoconjunctivitis).ab,ti.

48	exp infectious bovine rhinotracheitis/

49	(bovine adj1 rhinotracheitis).ab,ti.

50	exp bacillus anthracis/

51	bacillus anthracis.ab,ti.

52	exp bacillus cereus/

53	bacillus cereus.ab,ti.

54	exp Clostridium botulinum/

55	Clostridium botulinum.ab,ti.

56	exp Clostridium perfringens/

57	Clostridium perfringens.ab,ti.

58	exp listeriosis/

59	listeriosis.ab,ti.

60	exp methicillin-resistant staphylococcus aureus/

61	exp staphylococcus aureus/

62	staphylococcus aureus.ab,ti.

63	exp escherichia coli/

64	escherichia coli.ab,ti.

65	"e coli".ab,ti.

66	exp taenia saginata/

67	taenia saginata.ab,ti.

68	exp Toxoplasma gondii/

69	Toxoplasma gondii.ab,ti.

70	exp pathogens/

71	pathogen*.ab,ti.

72	exp infection/

73	infection*.ab,ti.

74	exp dermatomycoses/

75	dermatomycoses.ab,ti.

76	"ring worm*".ab,ti.

77	exp pestivirus/

78	pestivirus.ab,ti.

79	exp Bovine coronavirus/

80	"Bovine coronavirus".ab,ti.

81	exp Bovine leukemia virus/

82	"Bovine leukemia".ab,ti.

83	exp Neospora caninum/

84	"Neospora caninum".ab,ti.

85	exp Leptospira interrogans/

86	"Leptospira interrogans".ab,ti.

87	exp Campylobacter jejuni/

88	exp Campylobacter coli/

89	exp Mycobacterium bovis/

90	"Mycobacterium bovis".ab,ti.

91	exp Proteobacteria/

92	Proteobacteria.ab,ti.

93	exp bovine spongiform encephalopathy/

94	"bovine spongiform encephalopathy".ab,ti.

95	"mad cow disease*".ab,ti.

96	exp azithromycin/

97	azithromycin.ab,ti.

98	exp haematuria/

99	haematuria.ab,ti.

100	exp nematode infections/

101	(nematode* adj1 infect*).ab,ti.

102	exp bovine anaplasmosis/

103	"bovine anaplasmosis".ab,ti.

104	exp anthrax/

105	anthrax*.ab,ti.

106	exp sepsis/

107	sepsis.ab,ti.

108	exp otitis media/

109	"otitis media".ab,ti.

110	exp theileria parva/

111	"cattle fever".ab,ti.

112	"east coast fever".ab,ti.

113	exp theileriosis/

114	exp insect pests/

115	"insect pest* ".ab,ti.

116	exp metastigmata/

117	ticks.ab,ti.

118	(Acari adj1 Ixodidae).ab,ti.

119	exp amblyomma americanum/

120	"Amblyomma americanum".ab,ti.

121	"lone star tick* ".ab,ti.

122	exp Amblyomma maculatum/

123	"Amblyomma maculatum".ab,ti.

124	"gulf coast tick* ".ab,ti.

125	exp dermacentor variabilis/

126	"Dermacentor variabilis".ab,ti.

127	"american dog tick* ".ab,ti.

128	exp ixodes scapularis/

129	"Ixodes scapularis".ab,ti.

130	"deer tick* ".ab,ti.

131	"blacklegged tick* ".ab,ti.

132	exp vertebrate pests/

133	"vertebrate pest* ".ab,ti.

134	exp reproductive disorders/

135	"reproductive disorder*".ab,ti.

136	exp infertility/

137	infertility.ab,ti.

138	exp nutrient deficiencies/

139	"nutrient deficienc*".ab,ti.

140	(breed adj1 authentic*).ab,ti.

141	exp registration/

142	(breed adj1 registration).ab,ti.

143	(illegal adj1 drug*).ab,ti.

144	exp growth promoters/

145	"growth promoter*".ab,ti.

146	exp feed supplements/

147	(supplement adj1 feed*).ab,ti.

148	exp vitamins/

149	"vitamin*".ab,ti.

150	exp disease control/

151	exp disease surveys/

152	(disease adj1 manage*).ab,ti.

153	(disease* adj1 control*).ab,ti.

154	(disease* adj1 contain*).ab,ti.

155	(disease* adj1 survey*).ab,ti.

156	(disease* adj1 surveillance*).ab,ti.

157	exp vaccination/

158	exp vaccines/

159	"vaccination*".ab,ti.

160	"vaccine*".ab,ti.

161	exp disease prevention/

162	exp record keeping/

163	"record keep*".ab,ti.

164	exp emergencies/

165	emergency.ab,ti.

166	emergencies.ab,ti.

167	exp regulations/

168	"regulation*".ab,ti.

**169	or/1-168	[vet threats]**

170	exp veterinarians/

171	veterinar*.ab,ti.

172	exp veterinary practice/

173	exp veterinary services/

**174	or/170-173	[vet services]**

175	exp beef cattle/

176	(beef* adj1 cattle*).ab,ti.

177	cows/

178	cow*.ab,ti.

179	exp calves/

180	calve*.ab,ti.

181	calf*.ab,ti.

182	exp beef cows/

183	bulls/

184	bull*.ab,ti.

185	exp beef bulls/

186	exp heifers/

187	heifer*.ab,ti.

188	exp cattle/

189	cattle*.ab,ti.

190	veal/

191	veal*.ab,ti.

192	exp steers/

193	"steer*".ab,ti.

194	or/175-193

**195	194 not ("dairy cattle*" or "dairy cow*" or "dairy calf" or "dairy calve*").ab,ti. [beef cattle terms]**

**196	169 and 174 and 195**

**197	limit 196 to yr="1960 - 2024"**

**Number of Records Retrieved: 12,674**

# Search 3 - Genetics/Breed (Input Supply Chain Phase) Search Strategy

1	exp beef cattle/

2	(beef* adj1 cattle*).ab,ti.

3	cows/

4	cow*.ab,ti.

5	exp calves/

6	calve*.ab,ti.

7	calf*.ab,ti.

8	exp beef cows/

9	bulls/

10	bull*.ab,ti.

11	exp beef bulls/

12	exp heifers/

13	heifer*.ab,ti.

14	exp cattle/

15	cattle*.ab,ti.

16	veal/

17	veal*.ab,ti.

18	exp steers/

19	"steer*".ab,ti.

20	or/1-19

**21	20 not ("dairy cattle*" or "dairy cow*" or "dairy calf" or "dairy calve*").ab,ti. [beef cattle terms]**

22	exp cattle breeds/

23	(cattle adj1 breed*).ab,ti.

24	(beef adj2 breed*).ab,ti.

25	exp breeding programmes/

26	(cow* adj1 breed*).ab,ti.

27	exp genetics/

28	exp crossbreeding/

29	"crossbreed*".ab,ti.

30	exp heterosis/

31	Heterosis.ab,ti.

32	"Hybrid vigor".ab,ti.

33	"Genomic selection".ab,ti.

34	(gene* adj1 selection).ab,ti.

35	exp genetic variation/

**36	or/22-35	[breeding/genetics phase]**

37	exp genetic disorders/

38	exp hereditary diseases/

39	(gene* adj1 disorder*).ab,ti.

40	"hereditary disease* ".ab,ti.

41	"autosomal recessive disorder* ".ab,ti.

42	arachnomelia.ab,ti.

43	exp syndactyly/

44	"syndactyl*".ab,ti.

45	exp osteopetrosis/

46	osteopetrosis.ab,ti.

47	exp dwarfism/

48	dwarfism.ab,ti.

49	exp congenital abnormalities/

50	"crooked tail syndrome".ab,ti.

51	(muscular adj1 hyperplasia).ab,ti.

52	exp glycogenosis/

53	glycogenosis.ab,ti.

54	"glycogen storage disease".ab,ti.

55	exp porphyria/

56	Protoporphyria.ab,ti.

57	exp bone diseases/

58	"skeletal disorder* ".ab,ti.

59	(bone adj1 disease).ab,ti.

60	achondroplasia.ab,ti.

61	exp alopecia/

62	alopecia.ab,ti.

63	exp ankylosis/

64	Ankylosis.ab,ti.

65	(rigid adj1 joint*).ab,ti.

66	exp arthrogryposis/

67	arthrogryposis.ab,ti.

68	exp cryptorchidism/

69	cryptorchidism.ab,ti.

70	exp brachygnathia/

71	brachygnathia inferior.ab,ti.

72	"parrot mouth".ab,ti.

73	(ocular adj1 dermoid*).ab,ti.

74	exp double muscling/

75	"double muscling".ab,ti.

76	exp hypotrichosis/

77	hypotrichosis.ab,ti.

78	hairlessness.ab,ti.

79	"rat tail".ab,ti.

80	idiopathic epilepsy.ab,ti.

81	exp mannosidosis/

82	Mannosidosis.ab,ti.

83	exp maple syrup urine disease/

84	"maple syrup urine disease".ab,ti.

85	exp pigmentation disorders/

86	hypopigmentation.ab,ti.

87	exp polydactylia/

88	"Polydactyl*".ab,ti.

89	(extra adj1 toe*).ab,ti.

90	exp myeloencephalopathy/

91	myeloencephalopathy.ab,ti.

92	"weaver syndrome".ab,ti.

93	exp photosensitivity/

94	Photosensitivity.ab,ti.

95	tibial hemimelia.ab,ti.

96	(exotic adj1 germplasm).ab,ti.

97	(exotic adj1 breed*).ab,ti.

98	exp inbreeding/

99	"inbreed*".ab,ti.

100	(special* adj2 trait*).ab,ti.

101	(genetic adj1 introgression).ab,ti.

102	(cost adj1 genotyping).ab,ti.

103	exp malnutrition/

104	malnutrition.ab,ti.

105	exp reproductive disorders/

106	(reproduct* adj1 disorder*).ab,ti.

107	exp lupinus/

108	"lupine*".ab,ti.

109	Lupinus.ab,ti.

**110	or/37-109	[breeding/genetics threats]**

**111	21 and 36 and 110**

**112	limit 111 to yr="1960 - 2024"**

**Number of Records Retrieved: 5,576**

# Search 4 - Cow-Calf Operations (Production Supply Chain Phase) Search Strategy

1	exp beef cattle/

2	(beef* adj1 cattle*).ab,ti.

3	cows/

4	cow*.ab,ti.

5	exp calves/

6	calve*.ab,ti.

7	calf*.ab,ti.

8	exp beef cows/

9	bulls/

10	bull*.ab,ti.

11	exp beef bulls/

12	exp heifers/

13	heifer*.ab,ti.

14	exp cattle/

15	cattle*.ab,ti.

16	veal/

17	veal*.ab,ti.

18	exp steers/

19	"steer*".ab,ti.

20	or/1-19

21	**20 not ("dairy cattle*" or "dairy cow*" or "dairy calf" or "dairy calve*").ab,ti. [beef cattle terms]**

22	exp calf production/

23	exp beef production/

24	("cow calf" adj1 operation*).ab,ti.

25	(cow-calf adj1 operation*).ab,ti.

26	("cow calf" adj1 product*).ab,ti.

27	(cow-calf adj1 product*).ab,ti.

28	("cow calf" adj1 produce*).ab,ti.

29	(cow-calf adj1 produce*).ab,ti.

30	(cow-calf adj1 industr*).ab,ti.

31	("cow calf" adj1 industr*).ab,ti.

32	(calf adj1 manage*).ab,ti.

33	(calv* adj1 manage*).ab,ti.

34	(calf* adj product*).ab,ti.

35	(calv* adj1 product*).ab,ti.

36	(nutrition adj1 manage*).ab,ti.

37	(forage* adj1 manage*).ab,ti.

38	exp breeding season/

39	(breed* adj1 season*).ab,ti.

40	exp calving season/

41	(calving adj1 season*).ab,ti.

42	(calf* adj1 season*).ab,ti.

43	exp body condition/

44	"body condition".ab,ti.

**45	or/22-44	[cow-calf phase terms]**

46	exp age at weaning/

47	exp weaning/

48	exp weaning weight/

49	(wean* adj1 age).ab,ti.

50	(wean* adj1 weigh*).ab,ti.

51	exp birth weight/

52	(birth adj1 weigh*).ab,ti.

53	exp infant mortality/

54	exp maternal mortality/

55	exp stillbirths/

56	exp neonatal mortality/

57	exp perinatal mortality/

58	(infant* adj1 mortality).ab,ti.

59	(maternal adj1 mortality).ab,ti.

60	"stillbirth*".ab,ti.

61	(neonat* adj1 mortality).ab,ti.

62	(perinatal adj1 mortality).ab,ti.

63	"maternal parity".ab,ti.

64	exp breeding season/

65	"breed* season*".ab,ti.

66	"mating season*".ab,ti.

67	(birth* adj1 season*).ab,ti.

68	exp cattle diseases/

69	exp calf diseases/

70	exp brucellosis/

71	Brucellosis.ab,ti.

72	exp tuberculosis/

73	"Mycobacterium bovis".ab,ti.

74	exp viral diseases/

75	viral disease*.ab,ti.

76	exp mucosal disease/

77	mucosal disease*.ab,ti.

78	exp arboviruses/

79	arbovirus*.ab,ti.

80	exp Trypanosoma evansi/

81	Trypanosoma evansi.ab,ti.

82	exp leptospirosis/

83	leptospirosis.ab,ti.

84	exp bovine viral diarrhea virus 1/

85	"bovine viral diarrhea".ab,ti.

86	exp akabane virus/

87	akabane virus.ab,ti.

88	exp vibriosis/

89	vibriosis.ab,ti.

90	exp campylobacteriosis/

91	campylobacteriosis.ab,ti.

92	exp Enterobacteriaceae/

93	Enterobacteriaceae.ab,ti.

94	exp listeria monocytogenes/

95	listeria.ab,ti.

96	exp "foot and mouth disease"/

97	"foot and mouth disease*".ab,ti.

98	exp Salmonella/

99	Salmonella.ab,ti.

100 exp Campylobacter/

101 Campylobacter.ab,ti.

102 exp cysticercosis/

103 cysticercosis.ab,ti.

104 exp bovine herpesviruses/

105 (bovine adj1 herpesvirus*).ab,ti.

106 exp Bovine respiratory syncytial virus/

107 (Bovine adj1 respiratory).ab,ti.

108 exp bovine parainfluenza viruses/

109 "bovine parainfluenza".ab,ti.

110 exp infectious bovine keratoconjunctivitis/

111 (bovine adj1 keratoconjunctivitis).ab,ti.

112 exp infectious bovine rhinotracheitis/

113 (bovine adj1 rhinotracheitis).ab,ti.

114 exp bacillus anthracis/

115 bacillus anthracis.ab,ti.

116 exp bacillus cereus/

117 bacillus cereus.ab,ti.

118 exp Clostridium botulinum/

119 Clostridium botulinum.ab,ti.

120 exp Clostridium perfringens/

121 Clostridium perfringens.ab,ti.

122 exp listeriosis/

123 listeriosis.ab,ti.

124 exp methicillin-resistant staphylococcus aureus/

125 exp staphylococcus aureus/

126 staphylococcus aureus.ab,ti.

127 exp escherichia coli/

128 escherichia coli.ab,ti.

129 "e coli".ab,ti.

130 exp taenia saginata/

131 taenia saginata.ab,ti.

132 exp Toxoplasma gondii/

133 Toxoplasma gondii.ab,ti.

134 exp pathogens/

135 pathogen*.ab,ti.

136 exp infection/

137 infection*.ab,ti.

138 exp dermatomycoses/

139 dermatomycoses.ab,ti.

140 "ring worm*".ab,ti.

141 exp pestivirus/

142 pestivirus.ab,ti.

143 exp Bovine coronavirus/

144 "Bovine coronavirus".ab,ti.

145 exp Bovine leukemia virus/

146 "Bovine leukemia".ab,ti.

147 exp Neospora caninum/

148 "Neospora caninum".ab,ti.

149 exp Leptospira interrogans/

150 "Leptospira interrogans".ab,ti.

151 exp Campylobacter jejuni/

152 exp Campylobacter coli/

153 exp Mycobacterium bovis/

154 "Mycobacterium bovis".ab,ti.

155 exp Proteobacteria/

156 Proteobacteria.ab,ti.

157 exp bovine spongiform encephalopathy/

158 "bovine spongiform encephalopathy".ab,ti.

159 "mad cow disease*".ab,ti.

160 exp azithromycin/

161 azithromycin.ab,ti.

162 exp haematuria/

163 haematuria.ab,ti.

164 exp nematode infections/

165 nematode*.ab,ti.

166 exp bovine anaplasmosis/

167 "bovine anaplasmosis".ab,ti.

168 exp anthrax/

169 anthrax*.ab,ti.

170 exp sepsis/

171 sepsis.ab,ti.

172 exp otitis media/

173 "otitis media".ab,ti.

174 exp theileria parva/

175 "cattle fever".ab,ti.

176 "east coast fever".ab,ti.

177 exp theileriosis/

178 exp insect pests/

179 "insect pest* ".ab,ti.

180 exp metastigmata/

181 ticks.ab,ti.

182 (Acari adj1 Ixodidae).ab,ti.

183 exp amblyomma americanum/

184 "Amblyomma americanum".ab,ti.

185 "lone star tick* ".ab,ti.

186 exp Amblyomma maculatum/

187 "Amblyomma maculatum".ab,ti.

188 "gulf coast tick* ".ab,ti.

189 exp dermacentor variabilis/

190 "Dermacentor variabilis".ab,ti.

191 "american dog tick* ".ab,ti.

192 exp ixodes scapularis/

193 "Ixodes scapularis".ab,ti.

194 "deer tick* ".ab,ti.

195 "blacklegged tick* ".ab,ti.

196 exp paratuberculosis/

197 paratuberculosis.ab,ti.

198 "johne's disease".ab,ti.

199 exp sexually transmitted diseases/

200 (venereal adj1 disease*).ab,ti.

201 exp tritrichomonas foetus/

202 "tritrichomonas foetus".ab,ti.

203 "tritrichomonas fetus".ab,ti.

204 exp disease transmission/

205 exp disease vectors/

206 (disease adj1 vector*).ab,ti.

207 exp digestive disorders/

208 "digestive disorder* ".ab,ti.

209 "Frothy bloat".ab,ti.

210 exp cochliomyia hominivorax/

211 cochliomyia hominivorax.ab,ti.

212 vesicular stomatitis/

213 "vesicular stomatitis".ab,ti.

214 exp mosquito-borne diseases/

215 (mosquito adj2 disease*).ab,ti.

216 exp flea-borne diseases/

217 (flea* adj2 disease*).ab,ti.

218 "vampire bat* ".ab,ti.

219 exp tick-borne diseases/

220 exp rabies virus/

221 rabies.ab,ti.

222 exp babesia bovis/

223 "babesia bovis".ab,ti.

224 "texas cattle fever".ab,ti.

225 "shipping fever".ab,ti.

226 exp parasites/

227 (contaminat* adj1 vehicle*).ab,ti.

228 (contaminat* adj1 trailer*).ab,ti.

229 (dirty adj1 vehicle*).ab,ti.

230 exp hand washing/

231 "hand washing".ab,ti.

232 handwashing.ab,ti.

233 exp antibiotics/

234 "antibiotic*".ab,ti.

235 exp "transport of animals"/

236 "transport*".ab,ti.

237 exp quarantine/

238 "quarantine*".ab,ti.

239 exp accidents/

240 "accident*".ab,ti.

241 exp agricultural prices/

242 exp market prices/

243 (weak adj1 dollar*).ab,ti.

244 (corn adj1 price*).ab,ti.

245 (cattle adj1 inventory).ab,ti.

246 (cow* adj1 inventory).ab,ti.

247 (purchase adj1 price*).ab,ti.

248 (cost* adj1 cattle).ab,ti.

249 (cost* adj1 cow*).ab,ti.

250 (price adj1 variation).ab,ti.

251 (feed* adj1 price*).ab,ti.

252 (hay adj1 price*).ab,ti.

253 (pasture adj1 rent*).ab,ti.

254 exp drought/

255 "drought*".ab,ti.

256 (cold adj1 weather*).ab,ti.

257 exp climate change/

258 "climate change".ab,ti.

259 exp floods/

260 "flood*".ab,ti.

261 exp water quality/

262 exp water pollution/

263 (water adj1 quality).ab,ti.

264 (water adj1 pollut*).ab,ti.

265 (water adj1 contamin*).ab,ti.

266 exp soil quality/

267 (soil adj1 quality).ab,ti.

268 exp resource management/

269 (resource* adj1 manage*).ab,ti.

270 exp emissions/

271 emissions.ab,ti.

272 land degradation.sh.

273 (land adj1 degradation).ab,ti.

274 (pasture* adj1 deteriorat*).ab,ti.

275 exp natural disasters/

276 "natural disaster* ".ab,ti.

277 exp fire/

278 "fire*".ab,ti.

279 exp earthquakes/

280 "earthquake*".ab,ti.

281 "nuclear radiation* ".ab,ti.

**282 or/46-281	[cow-calf threat terms]**

**283 21 and 45 and 282**

**284 limit 283 to yr="1960 – 2024”**

**Number of Records Retrieved: 7,141**

# Search 5 - Backgrounder/Stocker Operations (Production Supply Chain Phase) Terms for Search Strategy

1	beef production/

2	(beef adj1 product*).ab,ti.

3	exp beef cattle/

4	(beef* adj1 cattle*).ab,ti.

5	cows/

6	cow*.ab,ti.

7	exp calves/

8	calve*.ab,ti.

9	calf*.ab,ti.

10	exp beef cows/

11	bulls/

12	bull*.ab,ti.

13	exp beef bulls/

14	exp heifers/

15	heifer*.ab,ti.

16	exp cattle/

17	cattle*.ab,ti.

18	veal/

19	veal*.ab,ti.

20	exp steers/

21	"steer*".ab,ti.

22	"stocker*".ab,ti.

23	or/1-22

**24	23 not ("dairy cattle*" or "dairy cow*" or "dairy calf" or "dairy calve*").ab,ti. [beef cattle terms]**

25	exp cattle farming/

26	backgrounder*.ab,ti.

27	backgrounding.ab,ti.

28	"stocker*".ab,ti.

29	exp auctions/

30	"auction*".ab,ti.

31	"direct sale* ".ab,ti.

32	"private treaty".ab,ti.

33	"retain* ownership ".ab,ti.

34	exp weaning/

35	(wean* adj1 manage*).ab,ti.

36	(nutrition adj1 manage*).ab,ti.

37	(feed* adj1 manage*).ab,ti.

38	exp risk management/

39	"risk management".ab,ti.

40	"preventive health".ab,ti.

41	"livestock risk protect*".ab,ti.

42	"grazing lease*".ab,ti.

**43	or/25-42	[stocker/backgrounder phase terms]**

44	exp mortality/

45	exp morbidity/

46	mortality.ab,ti.

47	morbidity.ab,ti.

48	exp weight gain/

49	(weight adj1 gain*).ab,ti.

50	exp selling prices/

51	exp profitability/

52	exp production costs/

53	"sale price* ".ab,ti.

54	"sell* price* ".ab,ti.

55	"profit*".ab,ti.

56	"product* cost*".ab,ti.

57	exp "cost benefit analysis"/

58	(cost* adj1 analysis).ab,ti.

59	"return on investment".ab,ti.

60	(feed* adj1 price*).ab,ti.

61	(truck* adj1 contamin*).ab,ti.

62	(transport* adj1 contamin*).ab,ti.

63	(data* adj1 secur*).ab,ti.

64	(data* adj1 breach*).ab,ti.

65	(pack* adj1 capacit*).ab,ti.

66	exp heat stress/

67	(heat adj1 stress*).ab,ti.

68	(stress* adj2 suscept*).ab,ti.

69	exp protozoal infections/

70	(protozoa* adj1 infect*).ab,ti.

71	(protozoa* adj1 disease*).ab,ti.

72	gall sickness.ab,ti.

73	exp bovine redwater fever/

74	redwater.ab,ti.

75	"red-water".ab,ti.

76	exp nutrient deficiencies/

77	(nutrient* adj1 deficienc*).ab,ti.

78	(nutrient* adj1 disorder*).ab,ti.

79	(nutrition* adj1 disorder*).ab,ti.

80	exp anthelmintics/

81	(anthelmintic* adj1 resist*).ab,ti.

82	"Cooperia (Nematoda)"/

83	cooperia.ab,ti.

84	exp Haemonchus/

85	Haemonchus.ab,ti.

86	(Gastrointest* adj1 distress*).ab,ti.

87	exp endotoxins/

88	"endotoxin*".ab,ti.

89	exp antibiotics/

90	"antibiotic*".ab,ti.

91	exp cattle diseases/

92	exp calf diseases/

93	exp brucellosis/

94	Brucellosis.ab,ti.

95	exp tuberculosis/

96	"Mycobacterium bovis".ab,ti.

97	exp viral diseases/

98	viral disease*.ab,ti.

99	exp mucosal disease/

100	mucosal disease*.ab,ti.

101	exp arboviruses/

102	arbovirus*.ab,ti.

103	exp Trypanosoma evansi/

104	Trypanosoma evansi.ab,ti.

105	exp leptospirosis/

106	leptospirosis.ab,ti.

107	exp bovine viral diarrhea virus 1/

108	"bovine viral diarrhea".ab,ti.

109	exp akabane virus/

110	akabane virus.ab,ti.

111	exp vibriosis/

112	vibriosis.ab,ti.

113	exp campylobacteriosis/

114	campylobacteriosis.ab,ti.

115	exp Enterobacteriaceae/

116	Enterobacteriaceae.ab,ti.

117	exp listeria monocytogenes/

118	listeria.ab,ti.

119	exp "foot and mouth disease"/

120	"foot and mouth disease*".ab,ti.

121	exp Salmonella/

122	Salmonella.ab,ti.

123	exp Campylobacter/

124	Campylobacter.ab,ti.

125	exp cysticercosis/

126	cysticercosis.ab,ti.

127	exp bovine herpesviruses/

128	(bovine adj1 herpesvirus*).ab,ti.

129	exp Bovine respiratory syncytial virus/

130	(Bovine adj1 respiratory).ab,ti.

131	exp bovine parainfluenza viruses/

132	"bovine parainfluenza".ab,ti.

133	exp infectious bovine keratoconjunctivitis/

134	(bovine adj1 keratoconjunctivitis).ab,ti.

135	exp infectious bovine rhinotracheitis/

136	(bovine adj1 rhinotracheitis).ab,ti.

137	exp bacillus anthracis/

138	bacillus anthracis.ab,ti.

139	exp bacillus cereus/

140	bacillus cereus.ab,ti.

141	exp Clostridium botulinum/

142	Clostridium botulinum.ab,ti.

143	exp Clostridium perfringens/

144	Clostridium perfringens.ab,ti.

145	exp listeriosis/

146	listeriosis.ab,ti.

147	exp methicillin-resistant staphylococcus aureus/

148	exp staphylococcus aureus/

149	staphylococcus aureus.ab,ti.

150	exp escherichia coli/

151	escherichia coli.ab,ti.

152	"e coli".ab,ti.

153	exp taenia saginata/

154	taenia saginata.ab,ti.

155	exp Toxoplasma gondii/

156	Toxoplasma gondii.ab,ti.

157	exp pathogens/

158	pathogen*.ab,ti.

159	exp infection/

160	infection*.ab,ti.

161	exp dermatomycoses/

162	dermatomycoses.ab,ti.

163	"ring worm*".ab,ti.

164	exp pestivirus/

165	pestivirus.ab,ti.

166	exp Bovine coronavirus/

167	"Bovine coronavirus".ab,ti.

168	exp Bovine leukemia virus/

169	"Bovine leukemia".ab,ti.

170	exp Neospora caninum/

171	"Neospora caninum".ab,ti.

172	exp Leptospira interrogans/

173	"Leptospira interrogans".ab,ti.

174	exp Campylobacter jejuni/

175	exp Campylobacter coli/

176	exp Mycobacterium bovis/

177	"Mycobacterium bovis".ab,ti.

178	exp Proteobacteria/

179	Proteobacteria.ab,ti.

180	exp bovine spongiform encephalopathy/

181	"bovine spongiform encephalopathy".ab,ti.

182	"mad cow disease*".ab,ti.

183	exp azithromycin/

184	azithromycin.ab,ti.

185	exp haematuria/

186	haematuria.ab,ti.

187	exp nematode infections/

188	nematode*.ab,ti.

189	exp bovine anaplasmosis/

190	"bovine anaplasmosis".ab,ti.

191	exp anthrax/

192	anthrax*.ab,ti.

193	exp sepsis/

194	sepsis.ab,ti.

195	exp otitis media/

196	"otitis media".ab,ti.

197	exp drought/

198	"drought*".ab,ti.

199	(cold* adj1 weather*).ab,ti.

200	exp climate change/

201	"climate chang*".ab,ti.

202	exp floods/

203	"flood*".ab,ti.

204	exp water quality/

205	exp water pollution/

206	(water adj1 quality).ab,ti.

207	(water adj1 pollut*).ab,ti.

208	(water adj1 contamin*).ab,ti.

209	exp soil quality/

210	(soil adj1 quality).ab,ti.

211	exp resource management/

212	(resource* adj1 manage*).ab,ti.

213	exp emissions/

214	emissions.ab,ti.

215	exp land degradation/

216	(land adj1 degradation).ab,ti.

217	(pasture* adj1 deteriorat*).ab,ti.

218	exp natural disasters/

219	"natural disaster* ".ab,ti.

220	exp fire/

221	"fire*".ab,ti.

222	exp earthquakes/

223	"earthquake*".ab,ti.

224	"nuclear radiation* ".ab,ti.

**225	or/44-224**

**226	24 and 43 and 225**

**227	limit 226 to yr="1960 – 2024”**

**Number of Records Retrieved: 6,162**

# Search 6 - Grass Finishing (Production Supply Chain Phase) Terms for Search Strategy

1	exp beef cattle/

2	(beef* adj1 cattle*).ab,ti.

3	cows/

4	cow*.ab,ti.

5	exp calves/

6	calve*.ab,ti.

7	calf*.ab,ti.

8	exp beef cows/

9	bulls/

10	bull*.ab,ti.

11	exp beef bulls/

12	exp heifers/

13	heifer*.ab,ti.

14	exp cattle/

15	cattle*.ab,ti.

16	veal/

17	veal*.ab,ti.

18	exp steers/

19	"steer*".ab,ti.

20	or/1-19

**21	20 not ("dairy cattle*" or "dairy cow*" or "dairy calf" or "dairy calve*").ab,ti. [beef cattle]**

22	exp cattle finishing/

23	(cattle adj1 finish*).ab,ti.

24	(cow* adj1 finish*).ab,ti.

25	(grass* adj1 finish*).ab,ti.

26	exp grassland management/

27	(pasture* adj1 manage*).ab,ti.

28	(grass* adj1 manage*).ab,ti.

29	exp stocking rate/

30	exp grazing intensity/

31	exp grazing time/

32	(stock* adj1 rate).ab,ti.

33	(graz* adj1 intensity).ab,ti.

34	(graz* adj1 time*).ab,ti.

35	(graz* adj1 day*).ab,ti.

36	exp supplementary feeding/

37	supplementation.ab,ti.

38	(forage* adj1 quality).ab,ti.

39	"fat cover".ab,ti.

40	exp body condition/

41	"body condition".ab,ti.

42	exp organic farming/

43	(organic adj1 beef).ab,ti.

44	"early maturity".ab,ti.

45	(lean adj1 beef).ab,ti.

**46	or/22-45	[grass finishing phase]**

47	exp market competition/

48	(market adj1 compet*).ab,ti.

49	exp plant protein/

50	(plant* adj2 protein*).ab,ti.

51	exp carbon footprint/

52	"carbon footprint".ab,ti.

53	exp methane/

54	(methane adj1 emissions).ab,ti.

55	exp international trade/

56	(international adj1 trade*).ab,ti.

57	(international adj1 market*).ab,ti.

58	exp world markets/

59	(world adj1 market*).ab,ti.

60	(international adj1 consumer*).ab,ti.

61	(international adj1 customer*).ab,ti.

62	exp irrigation/

63	"irrigation*".ab,ti.

64	exp land ownership/

65	(land adj1 sale*).ab,ti.

66	(land adj1 owner*).ab,ti.

67	(land adj2 turnover*).ab,ti.

68	exp certification/

69	(USDA adj1 certif*).ab,ti.

70	(USDA adj2 facilit*).ab,ti.

71	(USDA adj2 process*).ab,ti.

72	exp rangelands/

73	exp range management/

74	(range* adj1 fragment*).ab,ti.

75	(range* adj1 manage*).ab,ti.

76	(range* adj1 reduc*).ab,ti.

77	(range* adj1 quality).ab,ti.

78	exp pastures/

79	(pasture* adj1 quality).ab,ti.

80	(pasture* adj1 fragment*).ab,ti.

81	(pasture* adj1 manage*).ab,ti.

82	(pasture* adj1 reduc*).ab,ti.

83	exp "cost benefit analysis"/

84	(cost* adj1 benefit*).ab,ti.

85	"return on investment".ab,ti.

86	exp sustainability/

87	(sustain* adj1 farm*).ab,ti.

88	(sustain* adj1 ag*).ab,ti.

**89	or/47-88	[grass finishing threats]**

**90	21 and 46 and 89**

**91	limit 90 to yr="1960 - 2024"**

**Number of Records Retrieved: 7,496**

# Search 7- Feedlots (Production Supply Chain Phase) Search Strategy

1	exp beef/

2	beef*.ab,ti.

3	beef production/

4	(beef adj1 product*).ab,ti.

5	exp beef cattle/

6	(beef* adj1 cattle*).ab,ti.

7	cows/

8	cow*.ab,ti.

9	exp calves/

10	calve*.ab,ti.

11	calf*.ab,ti.

12	exp beef cows/

13	bulls/

14	bull*.ab,ti.

15	exp beef bulls/

16	exp heifers/

17	heifer*.ab,ti.

18	exp cattle/

19	cattle*.ab,ti.

20	veal/

21	veal*.ab,ti.

22	exp steers/

23	"steer*".ab,ti.

**24	or/1-23**

**25	24 not ("dairy cattle*" or "dairy cow*" or "dairy calf" or "dairy calve*").ab,ti. [beef cattle terms]**

26	exp feedlots/

27	"feedlot*".ab,ti.

28	(feed adj1 lot*).ab,ti.

29	(USDA adj1 "quality grade*").ab,ti.

30	("department of agriculture" adj1 "quality grade").ab,ti.

31	exp feed dispensers/

32	(feed* adj1 dispenser*).ab,ti.

33	"feeder*".ab,ti.

34	"days on feed".ab,ti.

35	(USDA adj1 "yield grade*").ab,ti.

36	(USDA adj1 grade*).ab,ti.

37	beta-agonists.ab,ti.

38	exp beta-adrenergic agonists/

39	exp growth promoters/

40	degree of finish.ab,ti.

41	"fat cover* ".ab,ti.

**42	or/26-41	[feedlot phase terms]**

43	exp animal welfare/

44	(animal* adj1 welfare*).ab,ti.

45	(animal* adj1 right*).ab,ti.

46	exp environmental policy/

47	(enviro* adj1 polic*).ab,ti.

48	(enviro* adj1 regulat*).ab,ti.

49	(enviro* adj1 impact*).ab,ti.

50	exp environmental impact/

51	exp consumer attitudes/

52	(consumer* adj1 attitude*).ab,ti.

53	(consumer* adj1 percept*).ab,ti.

54	(ammonia adj1 volatilization).ab,ti.

55	exp nitrogen fixation/

56	"Nitrogen fixation".ab,ti.

57	exp antibiotic resistance/

58	"Antimicrobial resistan*".ab,ti.

59	"antibiotic* resistan*".ab,ti.

60	(grain* adj1 price*).ab,ti.

61	(feed* adj1 price*).ab,ti.

62	exp ethanol/

63	"ethanol*".ab,ti.

64	(use* adj1 "feed grain*").ab,ti.

65	(feedlot* adj1 capacity).ab,ti.

66	exp theft/

67	theft.ab,ti.

68	(feed adj2 pathogen*).ab,ti.

69	exp vaccines/

70	(vet* adj1 vaccine*).ab,ti.

71	exp veterinary services/

72	(vet* adj1 service*).ab,ti.

73	(vet* adj1 access*).ab,ti.

74	exp cattle diseases/

75	exp calf diseases/

76	exp brucellosis/

77	Brucellosis.ab,ti.

78	exp tuberculosis/

79	"Mycobacterium bovis".ab,ti.

80	exp viral diseases/

81	viral disease*.ab,ti.

82	exp mucosal disease/

83	mucosal disease*.ab,ti.

84	exp arboviruses/

85	arbovirus*.ab,ti.

86	exp Trypanosoma evansi/

87	Trypanosoma evansi.ab,ti.

88	exp leptospirosis/

89	leptospirosis.ab,ti.

90	exp bovine viral diarrhea virus 1/

91	"bovine viral diarrhea".ab,ti.

92	exp akabane virus/

93	akabane virus.ab,ti.

94	exp vibriosis/

95	vibriosis.ab,ti.

96	exp campylobacteriosis/

97	campylobacteriosis.ab,ti.

98	exp Enterobacteriaceae/

99	Enterobacteriaceae.ab,ti.

100	exp listeria monocytogenes/

101	listeria.ab,ti.

102	exp "foot and mouth disease"/

103	"foot and mouth disease*".ab,ti.

104	exp Salmonella/

105	Salmonella.ab,ti.

106	exp Campylobacter/

107	Campylobacter.ab,ti.

108	exp cysticercosis/

109	cysticercosis.ab,ti.

110	exp bovine herpesviruses/

111	(bovine adj1 herpesvirus*).ab,ti.

112	exp Bovine respiratory syncytial virus/

113	(Bovine adj1 respiratory).ab,ti.

114	exp bovine parainfluenza viruses/

115	"bovine parainfluenza".ab,ti.

116	exp infectious bovine keratoconjunctivitis/

117	(bovine adj1 keratoconjunctivitis).ab,ti.

118	exp infectious bovine rhinotracheitis/

119	(bovine adj1 rhinotracheitis).ab,ti.

120	exp bacillus anthracis/

121	bacillus anthracis.ab,ti.

122	exp bacillus cereus/

123	bacillus cereus.ab,ti.

124	exp Clostridium botulinum/

125	Clostridium botulinum.ab,ti.

126	exp Clostridium perfringens/

127	Clostridium perfringens.ab,ti.

128	exp listeriosis/

129	listeriosis.ab,ti.

130	exp methicillin-resistant staphylococcus aureus/

131	exp staphylococcus aureus/

132	staphylococcus aureus.ab,ti.

133	exp escherichia coli/

134	escherichia coli.ab,ti.

135	"e coli".ab,ti.

136	exp taenia saginata/

137	taenia saginata.ab,ti.

138	exp Toxoplasma gondii/

139	Toxoplasma gondii.ab,ti.

140	exp pathogens/

141	pathogen*.ab,ti.

142	exp infection/

143	infection*.ab,ti.

144	exp dermatomycoses/

145	dermatomycoses.ab,ti.

146	"ring worm*".ab,ti.

147	exp pestivirus/

148	pestivirus.ab,ti.

149	exp Bovine coronavirus/

150	"Bovine coronavirus".ab,ti.

151	exp Bovine leukemia virus/

152	"Bovine leukemia".ab,ti.

153	exp Neospora caninum/

154	"Neospora caninum".ab,ti.

155	exp Leptospira interrogans/

156	"Leptospira interrogans".ab,ti.

157	exp Campylobacter jejuni/

158	exp Campylobacter coli/

159	exp Mycobacterium bovis/

160	"Mycobacterium bovis".ab,ti.

161	exp Proteobacteria/

162	Proteobacteria.ab,ti.

163	exp bovine spongiform encephalopathy/

164	"bovine spongiform encephalopathy".ab,ti.

165	"mad cow disease*".ab,ti.

166	exp azithromycin/

167	azithromycin.ab,ti.

168	exp haematuria/

169	haematuria.ab,ti.

170	exp nematode infections/

171	nematode*.ab,ti.

172	exp bovine anaplasmosis/

173	"bovine anaplasmosis".ab,ti.

174	exp anthrax/

175	anthrax*.ab,ti.

176	exp sepsis/

177	sepsis.ab,ti.

178	exp otitis media/

179	"otitis media".ab,ti.

180	exp theileria parva/

181	"cattle fever".ab,ti.

182	"east coast fever".ab,ti.

183	exp theileriosis/

184	exp insect pests/

185	"insect pest* ".ab,ti.

186	exp metastigmata/

187	ticks.ab,ti.

188	(Acari adj1 Ixodidae).ab,ti.

189	exp amblyomma americanum/

190	"Amblyomma americanum".ab,ti.

191	"lone star tick* ".ab,ti.

192	exp Amblyomma maculatum/

193	"Amblyomma maculatum".ab,ti.

194	"gulf coast tick* ".ab,ti.

195	exp dermacentor variabilis/

196	"Dermacentor variabilis".ab,ti.

197	"american dog tick* ".ab,ti.

198	exp ixodes scapularis/

199	"Ixodes scapularis".ab,ti.

200	"deer tick* ".ab,ti.

201	"blacklegged tick* ".ab,ti.

202	exp Mannheimia haemolytica/

203	"Mannheimia haemolytica".ab,ti.

204	"Pasteurella Haemolytica".ab,ti.

205	exp Mycoplasma bovis/

206	"Mycoplasma Bovis".ab,ti.

207	exp Histophilus somni/

208	"Histophilus somni".ab,ti.

209	exp polioencephalomalacia/

210	polioencephalomalacia.ab,ti.

211	exp drought/

212	"drought*".ab,ti.

213	(cold* adj1 weather*).ab,ti.

214	exp climate change/

215	"climate chang*".ab,ti.

216	exp floods/

217	"flood*".ab,ti.

218	exp water quality/

219	exp water pollution/

220	(water adj1 quality).ab,ti.

221	(water adj1 pollut*).ab,ti.

222	(water adj1 contamin*).ab,ti.

223	exp soil quality/

224	(soil adj1 quality).ab,ti.

225	exp resource management/

226	(resource* adj1 manage*).ab,ti.

227	exp emissions/

228	emissions.ab,ti.

229	exp land degradation/

230	(land adj1 degradation).ab,ti.

231	(pasture* adj1 deteriorat*).ab,ti.

232	exp natural disasters/

233	"natural disaster* ".ab,ti.

234	exp fire/

235	"fire*".ab,ti.

236	exp earthquakes/

237	"earthquake*".ab,ti.

238	"nuclear radiation* ".ab,ti.

**239	or/43-238	[feedlot threat terms]**

**240	25 and 42 and 239**

**241	limit 240 to yr="1960 - 2024"**

**Number of Records Retrieved: 3,505**

# Search 8 - Processing and Distribution Supply Chain Phase Search Strategy

1	exp beef/

2	beef*.ab,ti.

3	beef production/

4	(beef adj1 product*).ab,ti.

5	exp beef cattle/

6	(beef* adj1 cattle*).ab,ti.

7	cows/

8	cow*.ab,ti.

9	exp calves/

10	calve*.ab,ti.

11	calf*.ab,ti.

12	exp beef cows/

13	bulls/

14	bull*.ab,ti.

15	exp beef bulls/

16	exp heifers/

17	heifer*.ab,ti.

18	exp cattle/

19	cattle*.ab,ti.

20	veal/

21	veal*.ab,ti.

22	exp steers/

23	"steer*".ab,ti.

24	exp beefburgers/

25	exp canned meat/

26	exp cured meats/

27	exp dried meat/

28	exp frozen meat/

29	exp ground beef/

30	"ground beef".ab,ti.

31	exp steaks/

32	"steak*".ab,ti.

33	exp hamburgers/

34	"hamburger*".ab,ti.

35	(beef* adj1 hotdog*).ab,ti.

36	"brisket*".ab,ti.

37	exp meat cuts/

38	exp meat byproducts/

39	exp offal/

40	(meat adj2 byproduct*).ab,ti.

41	"offal*".ab,ti.

**42	or/1-41	[beef terms]**

43	exp slaughter/

44	"slaughter*".ab,ti.

45	exp food inspection/

46	exp carcasses/

47	exp carcass weight/

48	exp carcass yield/

49	exp carcass quality/

50	exp carcass composition/

51	"carcass*".ab,ti.

52	exp meat cuts/

53	(meat* adj1 cut*).ab,ti.

54	(prim* adj1 cut*).ab,ti.

55	exp meat grades/

56	(quality adj2 grad*).ab,ti.

57	exp packaging/

58	"packag*".ab,ti.

59	exp food storage/

60	exp refrigeration/

61	exp frozen storage/

62	exp cold storage/

63	storage.ab,ti.

64	(refrigerat* adj1 storage).ab,ti.

65	(cold* adj1 storage).ab,ti.

66	(frozen adj1 storage).ab,ti.

67	refrigeration.ab,ti.

68	exp refrigerated transport/

69	(refrigerat* adj1 transport*).ab,ti.

70	food delivery systems/

71	exp supermarkets/

72	"supermarket*".ab,ti.

73	(grocery adj1 store*).ab,ti.

74	exp retail prices/

75	exp food prices/

76	(retail adj1 price*).ab,ti.

77	(beef adj1 price*).ab,ti.

78	exp food safety/

79	(food adj1 safety).ab,ti.

80	exp information technology/

81	"information tech*".ab,ti.

82	"blockchain tech*".ab,ti.

83	exp regulations/

84	(regulat* adj1 compliance*).ab,ti.

85	HACCP.ab,ti.

86	cutability.ab,ti.

87	(dress* adj1 weigh*).ab,ti.

88	(grid* adj1 pric*).ab,ti.

89	(pric* adj1 contract*).ab,ti.

**90	or/43-89	[processing supply chain]**

91	exp bovine spongiform encephalopathy/

92	bovine spongiform encephalopathy.ab,ti.

93	exp Salmonella/

94	salmonella.ab,ti.

95	exp foodborne diseases/

96	(foodborne adj1 disease*).ab,ti.

97	(foodborn* adj1 disease*).ab,ti.

98	(foodborn* adj1 bacteria*).ab,ti.

99	(foodborne adj1 bacteria*).ab,ti.

100	exp campylobacteriosis/

101	Campylobacteriosis.ab,ti.

102	exp Campylobacter/

103	campylobacter.ab,ti.

104	exp Shiga toxin-producing Escherichia coli/

105	Escherichia coli.ab,ti.

106	"e coli".ab,ti.

107	(enteric adj1 pathogen*).ab,ti.

108	(shiga adj1 toxin*).ab,ti.

109	exp cysticercosis/

110	Cysticercosis.ab,ti.

111	exp Taenia solium/

112	Taenia solium.ab,ti.

113	exp Taenia saginata/

114	Taenia saginata.ab,ti.

115	exp Toxoplasma gondii/

116	Toxoplasma gondii.ab,ti.

117	exp congenital toxoplasmosis/

118	congenital toxoplasmosis.ab,ti.

119	exp Staphylococcus aureus/

120	Staphylococcus aureus.ab,ti.

121	"country of origin label".ab,ti.

122	(beef adj1 crime*).ab,ti.

123	(food adj1 integrity).ab,ti.

124	(beef adj1 fraud).ab,ti.

125	(food adj1 fraud).ab,ti.

126	exp labelling/

127	(label* adj1 fraud*).ab,ti.

128	"mislabel*".ab,ti.

129	(incorrect* adj1 label*).ab,ti.

130	"counterfeit*".ab,ti.

131	exp adulteration/

132	adulteration.ab,ti.

133	"tamper*".ab,ti.

134	exp food colourants/

135	(illegal adj1 colourant*).ab,ti.

136	(illegal adj1 colorant*).ab,ti.

137	(colourant* adj1 additive*).ab,ti.

138	exp food additives/

139	(chemical* adj1 additive*).ab,ti.

140	(aroma* adj1 additive*).ab,ti.

141	(flavor* adj1 additive*).ab,ti.

142	exp bulking agents/

143	(bulk* adj1 agent*).ab,ti.

144	(date* adj1 tamper*).ab,ti.

145	(label* adj1 tamper*).ab,ti.

146	(date adj1 chang*).ab,ti.

147	"spoiled product*".ab,ti.

148	(short adj1 weigh*).ab,ti.

149	exp carbon footprint/

150	"carbon* footprint*".ab,ti.

151	(truck* adj1 contamin*).ab,ti.

152	(transport* adj1 contamin*).ab,ti.

153	(border* adj1 inspect*).ab,ti.

154	(border* adj1 reject*).ab,ti.

155	(heavy metal adj1 accumulat*).ab,ti.

156	(carcass* adj1 contamin*).ab,ti.

157	"public perception*".ab,ti.

158	(consumer* adj1 percept*).ab,ti.

159	consumer awareness.ab,ti.

160	(consumer* adj1 aware*).ab,ti.

161	(waste adj2 dispos*).ab,ti.

162	(supply adj2 disrupt*).ab,ti.

163	(commerce* adj1 loop*).ab,ti.

164	(packag* adj1 inventory).ab,ti.

165	(process* adj1 reduc*).ab,ti.

166	exp safety at work/

167	(occupation* adj1 safety).ab,ti.

168	(work* adj1 safety).ab,ti.

169	(safety adj1 hazard*).ab,ti.

170	exp occupational hazards/

171	(worker* adj2 strike*).ab,ti.

172	(labor* adj1 dispute*).ab,ti.

173	(labour* adj1 dispute*).ab,ti.

174	exp floods/

175	"flood*".ab,ti.

176	exp natural disasters/

177	"natural disaster*".ab,ti.

178	exp fire/

179	"fire*".ab,ti.

180	exp earthquakes/

181	"earthquake*".ab,ti.

182	"nuclear radiation".ab,ti.

183	"solar flare* ".ab,ti.

184	(severe adj1 weather).ab,ti.

185	"cyber attack* ".ab,ti.

186	(computer* adj1 virus*).ab,ti.

187	(industrial adj1 espionage).ab,ti.

188	"cyber security".ab,ti.

189	"power outage*".ab,ti.

190	"fuel shortage*".ab,ti.

191	"electricity outage*".ab,ti.

192	"gas outage*".ab,ti.

193	(material* adj1 disruption*).ab,ti.

194	(transport* adj1 network*).ab,ti.

195	(temp* adj1 control*).ab,ti.

**196	or/91-195	[processing threat terms]**

**197	42 and 90 and 196**

**198	limit 197 to yr="1960 - 2024"**

**Number of Records Retrieved: 12,075**

# Search 9 - Marketing and Consumers Supply Chain Phase Terms for Search Strategy

1	exp beef/

2	beef*.ab,ti.

3	beef production/

4	(beef adj1 product*).ab,ti.

5	veal/

6	veal*.ab,ti.

7	exp beefburgers/

8	exp canned meat/

9	exp cured meats/

10	exp dried meat/

11	exp frozen meat/

12	exp ground beef/

13	"ground beef".ab,ti.

14	exp steaks/

15	"steak*".ab,ti.

16	exp hamburgers/

17	"hamburger*".ab,ti.

18	(beef* adj1 hotdog*).ab,ti.

19	"brisket*".ab,ti.

20	exp meat cuts/

21	exp meat byproducts/

22	exp offal/

23	(meat adj2 byproduct*).ab,ti.

24	"offal*".ab,ti.

**25	or/1-24	[beef product terms]**

26	exp consumer behaviour/

27	exp consumer preferences/

28	exp consumer attitudes/

29	exp consumer expenditure/

30	exp consumer prices/

31	exp consumers/

32	"consumer*".ab,ti.

33	exp willingness to pay/

34	(willing* adj2 pay*).ab,ti.

35	(willing* adj2 buy).ab,ti.

36	exp marketing/

37	exp labelling/

38	marketing.ab,ti.

39	labelling.ab,ti.

40	labels.ab,ti.

**41	or/26-40	[marketing/consumer terms]**

42	exp market competition/

43	(market adj1 volatil*).ab,ti.

44	(market* adj1 compet*).ab,ti.

45	exp markets/

46	exp marketing margins/

47	(market* adj1 margin*).ab,ti.

48	(price* adj1 spread*).ab,ti.

49	exp prices/

50	exp market prices/

51	pricing.ab,ti.

52	(price* adj1 spread*).ab,ti.

53	(price* adj1 discover*).ab,ti.

54	(prices* adj1 transpar*).ab,ti.

55	(cash* adj1 market*).ab,ti.

56	(purchas* adj1 power*).ab,ti.

57	exp data processing/

58	(data* adj1 process*).ab,ti.

59	(data* adj1 manage*).ab,ti.

60	exp epidemics/

61	exp pandemics/

62	"epidemic*".ab,ti.

63	"pandemic*".ab,ti.

64	exp packaging/

65	"packag*".ab,ti.

66	(pack* adj1 capacity).ab,ti.

67	exp working conditions/

68	exp labour market/

69	(work* adj1 condition*).ab,ti.

70	(labor* adj1 condition*).ab,ti.

71	(labour* adj1 condition*).ab,ti.

72	(labor* adj1 market*).ab,ti.

73	(labour* adj1 market*).ab,ti.

74	(worker* adj1 compensat*).ab,ti.

75	"wage*".ab,ti.

76	"salar*".ab,ti.

77	(consumer* adj2 confiden*).ab,ti.

78	(consumer* adj2 trust*).ab,ti.

79	exp foodborne diseases/

80	exp food safety/

81	(foodborne* adj1 disease*).ab,ti.

82	(foodborn* adj1 disease*).ab,ti.

83	(foodborne* adj1 illness*).ab,ti.

84	(foodborn* adj1 illness*).ab,ti.

85	(foodborne* adj1 pathogen*).ab,ti.

86	(foodborn* adj1 pathogen*).ab,ti.

87	(food* adj1 sick*).ab,ti.

88	(food* adj1 safety).ab,ti.

89	(risk* adj1 percept*).ab,ti.

90	exp Salmonella/

91	salmonella.ab,ti.

92	exp botulism/

93	botulism.ab,ti.

94	exp Escherichia coli/

95	Escherichia coli.ab,ti.

96	"e coli".ab,ti.

97	exp food contamination/

98	"contaminat*".ab,ti.

**99	or/42-98	[marketing/consumer threats]**

**100	25 and 41 and 99**

**101	limit 100 to yr="1960 - 2024"**

**Number of Records Retrieved: 3,746**

# Search 10 - “Other” Supply Chain Phase Terms for Search Strategy

1	exp beef/

2	beef*.ab,ti.

3	beef production/

4	(beef adj1 product*).ab,ti.

5	exp beef cattle/

6	(beef* adj1 cattle*).ab,ti.

7	exp beef cows/

8	exp beef bulls/

9	exp ground beef/

10	"ground beef".ab,ti.

11	exp steaks/

12	"steak*".ab,ti.

13	exp hamburgers/

14	"hamburger*".ab,ti.

15	(beef* adj1 hotdog*).ab,ti.

16	"brisket*".ab,ti.

17	exp meat cuts/

18	exp meat byproducts/

19	exp offal/

20	(meat adj2 byproduct*).ab,ti.

21	"offal*".ab,ti.

**22	or/1-21	[beef production terms - focused on “beef”]**

23	exp agricultural manpower/

24	exp labour market/

25	exp migrant labour/

26	exp farm workers/

27	(ag* adj1 labour*).ab,ti.

28	(ag* adj1 labor*).ab,ti.

29	(farm* adj1 labour*).ab,ti.

30	(farm* adj1 labor*).ab,ti.

31	(farm* adj1 work*).ab,ti.

32	(migrant* adj1 labor*).ab,ti.

33	(migrant* adj1 labour*).ab,ti.

34	(migrant* adj1 work*).ab,ti.

35	(ag* adj1 work*).ab,ti.

36	exp strikes/

37	"strike*".ab,ti.

38	(labor* adj1 short*).ab,ti.

39	(labour* adj1 short*).ab,ti.

40	exp data processing/

41	exp information technology/

42	exp internet/

43	exp cybercrime/

44	(data* adj1 process*).ab,ti.

45	(informat* adj1 tech*).ab,ti.

46	cybercrime.ab,ti.

47	"cybertech*".ab,ti.

48	(data* adj1 breech*).ab,ti.

49	(security adj1 breech*).ab,ti.

50	(info* adj1 privacy).ab,ti.

51	(cyber adj1 attack*).ab,ti.

52	(comput* adj1 virus*).ab,ti.

53	(indust* adj1 espion*).ab,ti.

54	exp electricity/

55	electricity.ab,ti.

56	exp telecommunications/

57	exp digital technology/

58	(grid* adj1 condition*).ab,ti.

59	"telecommunicat*".ab,ti.

60	(wireless adj1 service*).ab,ti.

61	(cell* adj1 phone*).ab,ti.

62	exp electric power/

63	(power adj1 outage*).ab,ti.

64	(electric* adj1 outage*).ab,ti.

65	(power* adj1 loss*).ab,ti.

66	(electric* adj1 loss*).ab,ti.

67	(logistic* adj1 disrupt*).ab,ti.

68	("supply chain" adj1 disrupt*).ab,ti.

69	(fuel* adj1 short*).ab,ti.

70	(gas adj1 shortage*).ab,ti.

71	(fuel adj1 emergenc*).ab,ti.

72	(facilit* adj1 loss*).ab,ti.

73	(infrastructure* adj1 fail*).ab,ti.

74	(environment* adj1 disaster*).ab,ti.

75	exp taxes/

76	taxes.ab,ti.

77	(trade* adj1 relation*).ab,ti.

78	exp trade relations/

79	exp world markets/

80	exp international trade/

81	(world adj1 market*).ab,ti.

82	(world adj1 trade*).ab,ti.

83	(internation* adj1 market*).ab,ti.

84	(internation* adj1 trade*).ab,ti.

85	(USDA adj1 certif*).ab,ti.

86	(USDA adj1 standard*).ab,ti.

87	("department of ag*" adj1 certif*).ab,ti.

88	("department of ag*" adj1 standard*).ab,ti.

89	(internation* adj1 standard*).ab,ti.

90	(internation* adj2 certif*).ab,ti.

91	(nation* adj2 certif*).ab,ti.

92	exp ethanol production/

93	"ethanol*".ab,ti.

94	(meat* adj1 alternat*).ab,ti.

95	("plant based" adj1 meat*).ab,ti.

96	("cell based" adj1 meat*).ab,ti.

97	(artificial adj1 meat*).ab,ti.

98	(culture* adj1 meat*).ab,ti.

**99	or/23-98	[threats for across the beef supply chain]**

**100	22 and 99**

**101	limit 100 to yr="1960 - 2024"**

**Number of Records Retrieved: 3,352**
