# Supplementary material for: Systematic mapping of threats to U.S. beef production
Source: Transl Anim Sci. 2026 Sep 21;10:txag143. doi: 10.1093/tas/txag143 (PMC13677081; doi:10.1093/tas/txag143)
Supplement: Supplementary file 1 [file txag143_supplementary_data.zip › Supplementary material B.docx]

**Guidebook of Interactive Evidence Gap Maps**

**1. General introduction of Evidence Gap Maps**

EPPI-Mapper is an application that creates interactive evidence gap maps (EGMs). Developed by the EPPI-Centre in partnership with the Campbell Collaboration. The first version was created in early 2018. It required studies to be coded in EPPI-Reviewer first. EPPI mapper is an interactive tool that uses EPPI reviewer data and enables the exploration of a body of evidence by mapping collective literature through three or more dimensions, specifying the data to be included in columns, rows, and within the cell. The map generated from EPPI-Mapper exists as a standalone HTML file. The map can be customized and published online. Evidence (gap) maps are visual ways to share the research findings. Evidence gap maps are visual presentations of evidence or lack of evidence in a domain. They allow easy identification of where and how research has been conducted, as well as the research gaps that exist. By clicking on any cell, the “reader” is opened, displaying the items that have a particular cross-section of data. Each intersection point indicates the number of corresponding records (e.g. the studies). The number of records in each node can be broken down into categories and represented as bubbles.

**2. Layout and style**

***Supplementary Figure 1.* *Map layout***

The menu contains many options like *Filters*, *Hide Headers*, *Full Screen*, *View Records*, and *Accessibility*. *Hide headers* allow you to hide the header areas at the top of the screen, while *full screen* allows the browser to fill your entire screen. *View Records* means all studies in the map will be listed. As seen from Supplementary Figure 1, each row and column has sections and subsections. Under article content fields, there are subsections like veterinary and animal science, food safety, microbiology, etc. *Style* can be pre-set or changed. As you click the icons on the right of the menu, the available styles are bubble, heat, mosaic, donut, and text (see Supplementary Figure 2). Most maps have some type of filtering mechanism for a further breakdown of coding. Maps can be filtered depending on the data behind it. You can click “Filters” on the left of the menu to filter based on segment. As demonstrated in Supplementary Figure 3, you can filter by the threat type. If you only want to see the accidental/non-targeted threat type, you can click, and the map will only present articles that fit those criteria.

| Bubble | Heat | Mosaic | Donut | Text |
| --- | --- | --- | --- | --- |
|  |  |  |  |  |

***Supplementary Figure 2. Map style***

***Supplementary Figure 3. Map Filter***

Another feature is the “*View Records,”* which lets you filter through certain variables in the rows/columns. You can group by option (none/segment). Detailed information on the study is displayed on the right. You can sort/filter by title, authors, and abstract. At the bottom of the study information, you can click and download the chosen manuscript/study. The Records panel has a “Download Listed References” button. As you finish your filter process, you can click the “Download Listed References” to generate an RIS formatted file of the listed records. If you check all the boxes, you will be able to download the entire dataset (1112 records). If you select only the references of interest in a search, you can download the references based on your selection (see Supplementary Figure 4). For example, if you are only interested in studies that focus on veterinary service in the production phase, you can check the veterinary service box and uncheck the rest of the boxes under the production phase. It will result in 429 records, and you will be able to download the 429 records by clicking the “Download Listed References button.” After downloading the RIS file, you can view the record in Covidence or other reference manager software. RIS is a ‘standard’ format for references that can be used with all reference management software (e.g., EndNote). Another option is to click on a cell/the cross-section of the row and column to open the references panel. You can click the cell (hover to display) to open the reference panel. Codes selected on the left can be adjusted (see Supplementary Figure 5).

***Supplementary Figure 4. View records***

***Supplementary Figure 5. Reference of the record***

**3. Three interactive maps from the Beef supply chain project**

3.1 Map 1 threats map

Each interactive map includes three selected variables, with columns, rows, and different bubble colors representing the subcategories of each variable. The size of the bubble represents the number of studies included.

***Supplementary Figure 6. Overview of Map 1 threat map***

Map 1 (Supplementary Figure 6) includes the production phase as the column, threat categories as the row, and the color of the bubble represents different threat types. As seen from Map 1, studies identify biological threats as the most prevalent.

3.2 Map 2 disease map

***Supplementary Figure 7. Overview of Map 2 disease map***

Map 2 (Supplementary Figure 7) includes the production phase as the column, specific diseases as the row, and the color of the bubble represents threat impacts. As demonstrated in Map 2, research is primarily focused on bovine respiratory disease (BRD), bovine viral diarrhea, parasite infections, and foot and mouth disease (FMD).

3.3 Map 3 solution map

Map 3 includes threat categories as the row, solution as the column, and the color of the bubbles represent the effectiveness of the solution. As shown in Map 3 (Supplementary Figure 8), solutions are focused on diagnostic and surveillance programs, vaccination and disease control, as well as environmental and management practices.

***Supplementary Figure 8. Overview of Map 3 solution map***

3.4 An example of how to identify an article

Supplementary Figure 9 provides an example from Map 3 of how to identify an article of your interest. Through this step-by-step guidance, you can access your article from the link embedded. O'Connor et al. (2019) explored the effect of the licensed *Moraxella bovoculi* vaccine on infectious bovine keratoconjunctivitis (IBK). The color represents the effectiveness of the solution. Here, orange represents not significant, indicating the effect of the vaccine was not significant.

|   1. Click the cross-section of biological from threat categories and vaccination and disease control from solutions. |   2.230 studies/records were identified. |
| --- | --- |
|   3. The third study looks interesting; click that, scroll to the bottom, and click the blue link. |   4. The link will take you to the article's publication website. |

***Supplementary Figure 9. Process of identifying one article of your interest***

a. Acknowledgement. '"This material is based upon work supported by the U.S. Department of Homeland Security under Grant Award Number, Award No 18STCBT00001 through the Cross-Border Threat Screening and Supply Chain Defense Center of Excellence.

b. Disclaimer. "The views and conclusions contained in this document are those of the authors and should not be interpreted as necessarily representing the official policies, either expressed or implied, of the U.S. Department of Homeland Security."
